# The myeloid IL-1 receptor limits IL-27–mediated endothelial type I IFN during nephrotoxic serum nephritis

**DOI:** 10.1172/jci.insight.200728

**Published:** 2026-09-01

**Authors:** Yanting Chen, Yu Li, Jiafa Ren, Chia-Chun Wu, Xiaohan Lu, Achintya Inumarty, Steven D. Crowley, Jamie R. Privratsky

**Affiliations:** 1 Department of Anesthesiology, Department of Medicine, Duke University Medical Center, Durham, North Carolina, USA.; 2 Department of Anesthesiology, Shanxi Province Cancer Hospital/Shanxi Hospital Affiliated to Cancer Hospital, Chinese Academy of Medical Sciences/Cancer Hospital Affiliated to Shanxi Medical University, China.; 3 Division of Nephrology, Department of Medicine, Duke University Medical Center, Durham, North Carolina, USA.; 4 Division of Nephrology, Department of Internal Medicine, Chi Mei Medical Center, Tainan, Taiwan.; 5 Durham VA Medical Center, Durham, North Carolina, USA.; 6 Department of Pharmacology and Cancer Biology, Duke University Medical Center, Durham, North Carolina, USA.

**Keywords:** Immunology, Inflammation, Nephrology, Chronic kidney disease, Cytokines, Macrophages

## Abstract

Autoimmune kidney diseases can cause glomerulonephritis and tubulointerstitial nephritis, which if unresolved, lead to progressive glomerulosclerosis and tubulointerstitial fibrosis. The IL-1 receptor (IL-1R1) is known to have divergent and cell-specific effects in kidney injury. We hypothesized that IL-1R1 would dampen pro-inflammatory activation of myeloid cells such that deletion of myeloid cell IL-1R1 would exacerbate autoimmune nephritis. Mice with myeloid cell–specific deletion of IL-1R1 (*LysMCre^+^/Il1r1^fl/fl^*, hereafter MKO) and littermate controls (*LysMCre^–^/Il1r1^fl/fl^*, MWT) were subjected to nephrotoxic serum (NTS) nephritis. MKO mice demonstrated worsened glomerular and tubular injury as indicated by increased albuminuria, glomerular injury scores, and kidney mRNA levels of kidney injury molecule 1 (KIM-1) (*Havcr1*) and neutrophil gelatinase-associated lipocalin (NGAL/*Lcn2*). We further found that myeloid IL-1R1 deficiency resulted in increased myeloid cell ER stress and expression of the heterodimeric cytokine *Ebi3/Il27a* (IL-27). IL-27 then induced increased type I IFN expression by kidney endothelial cells. In turn, anti–IL-27 limited type I IFN expression in endothelial cells and NTS nephritis, and anti-IFNAR1 therapy ameliorated glomerular and tubular injury in MKO mice. Thus, we demonstrated a myeloid cell/endothelial cell immunoregulatory axis whereby myeloid IL-1R1 activity constrained endothelial type I IFN generation to limit chronic kidney damage.

## Abstract 2

We identify a myeloid cell-endothelial cell immunoregulatory axis whereby myeloid IL-1R1 activity constrained endothelial type I IFN generation to limit autoimmune kidney damage

## Graphical abstract

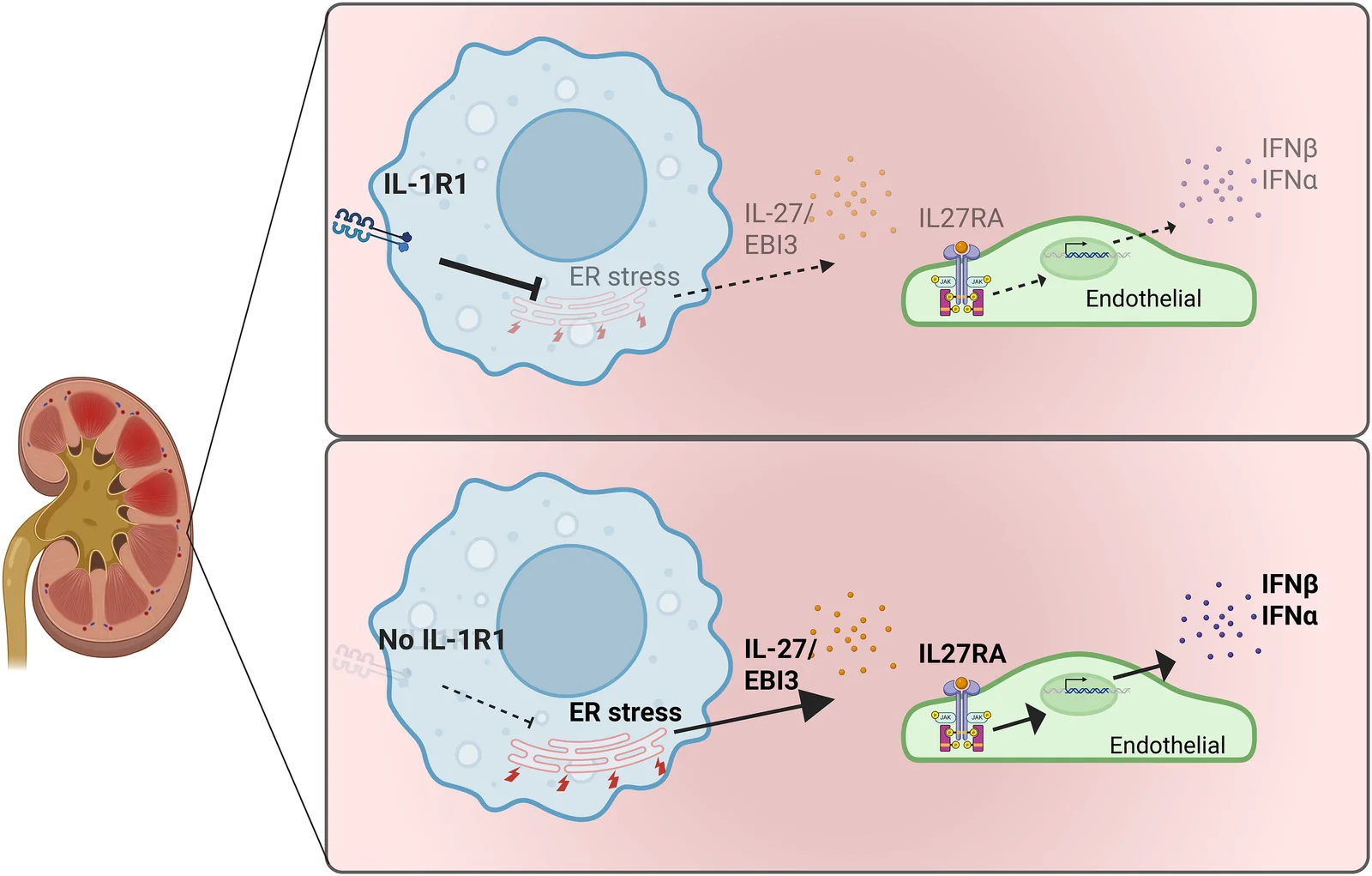

## Introduction

Chronic kidney disease (CKD) affects more than 850 million people worldwide (1). Clinically, CKD is characterized by a prolonged and progressive decline in kidney function, measured by a decreased glomerular filtration rate and the presence of albuminuria and tubulointerstitial damage (2). During CKD progression, injury to glomerular components and unresolved kidney tubule cell injury lead to progressive glomerulosclerosis and tubulointerstitial fibrosis resulting in nephron loss (3, 4). CKD can be caused by multiple insults including acute kidney injury (AKI), diabetes mellitus, toxins, genetic diseases, and autoimmune diseases. Of autoimmune diseases, some of the most common to affect the kidney include systemic lupus erythematosus, which causes lupus nephritis; antineutrophilic cytoplasmic autoantibody vasculitis; and antibody-mediated kidney diseases such as anti-glomerular basement membrane (GBM) disease, all of which lead to glomerulosclerosis and/or tubulointerstitial nephritis. The processes contributing to progressive CKD in autoimmune kidney diseases involve proinflammatory cytokines, cellular stress, and aberrant immune responses with dysregulated tissue repair (5, 6).

During kidney inflammation, cytokines play important, complex roles in modulating the cell-cell interactions that regulate kidney healing. For instance, IL-1 (IL-1α and IL-1β) isoforms bind to their cell surface receptor, IL-1R1, and can exacerbate kidney damage (7–13). However, IL-1R1 on certain cell types, including myeloid cells, can actually limit inflammation and kidney injury (12, 14, 15) implying cell-specific contextual roles for IL-1R1 in the inflammatory response. Similarly, the heterodimeric cytokine IL-27, made up of the subunits Epstein-Barr virus–induced gene 3 (EBI3/*Ebi3*) and IL-27p28 (*Il27a*), has pleiotropic and sometimes opposing effects on different cell types during inflammation (16). IL-27 has been highly studied in the context of its effects on lymphocytes where it can modulate Th differentiation responses (17, 18). However, IL-27 also increases expression of IFN-stimulated genes in multiple cell types (19–23). Type I IFNs (i.e., IFN-α, IFN-β) play a protective role in the host antiviral response (24, 25) and antitumor immune response (26). However, persistent type I IFN production can drive chronic inflammation, especially in the kidney, where type I IFNs have been highly implicated in driving the glomerular and tubulointerstitial lesions that cause lupus nephritis, CKD, glomerulosclerosis, and viral nephropathies (27–30). Thus, there is an important need to identify cellular sources and mechanisms to harness the protective effects of these cytokines during kidney immune responses while limiting their detrimental effects.

Myeloid cells play crucial roles in the kidney immune response. During infection or injury, myeloid cells (macrophages, monocytes, DCs, and neutrophils) are the first responders and release inflammatory mediators and cytokines such as IL-1β, TNF, and type I IFN to initiate the immune response (31). However, myeloid cells are also critical to clear invading microorganisms and cellular debris to promote repair of neighboring cells. Indeed, all myeloid cells can play protective or detrimental effects depending on their phenotype/polarization state, the type of kidney injury, and the timing after injury (32–37). Beyond myeloid cells, endothelial cells also play a prominent role in the kidney inflammatory response as they regulate passage of myeloid cells and other immune cells to sites of injury. In the kidney, endothelial cells form the glomerular filtration barrier with podocytes and the basement membrane and thus are exposed to circulating immune complexes and cytokines (30). As such, the kidney, and particularly the glomerulus, is often the initial site of injury in autoimmune diseases. We and others have previously demonstrated that myeloid cells and endothelial cells regulate each other’s functions during kidney injury, including in response to inflammatory cytokines (38, 39). Thus, therapeutic strategies targeting inflammatory mediators that regulate kidney myeloid cell–endothelial cell interactions could have great potential to limit chronic kidney damage and fibrosis development.

In this study, we aim to further investigate the protective role of myeloid IL-1R1 during the progression of autoimmune kidney injury. Based on our previous studies where we found a protective antiinflammatory role of IL-1R1 on CD11c^+^ myeloid cells during ischemic AKI (14), we hypothesized that deletion of myeloid IL-1R1 would promote inflammation and exacerbate autoimmune nephritis. In this study, we showed that myeloid IL-1R1 limits glomerulonephritis through a mechanism modulating myeloid ER stress and EBI3/IL-27 expression, which in turn reduces endothelial type I IFN generation.

## Results

### Deletion of IL-1R1 in LysM^+^ myeloid cells worsens chronic kidney injury after nephrotoxic serum nephritis.

Nephrotoxic serum (NTS) nephritis is a commonly used murine model of immune-mediated kidney injury that utilizes timed injections of sheep IgG and anti-GBM antibody to induce both glomerular and tubular damage, effects that are most pronounced on the 129/SvEv background (40). To determine the role of IL-1R1 in myeloid cells after NTS, we employed a genetic model to delete IL-1R1 from LysM^+^ cells in mice on the 129/SvEv background (*LysMCre^+^/Il1r1^fl/fl^*, hereafter MKO) (Supplemental Figure 1A; supplemental material available online with this article; https://doi.org/10.1172/jci.insight.200728DS1). We confirmed that, compared with (*LysMCre^–^/Il1r1^fl/fl^*, hereafter MWT) mice, *Il1r1* was specifically deleted from peritoneal macrophages in MKO mice, whereas expression was preserved in other tissues (Supplemental Figure 1, B–E). We then subjected MWT and MKO mice to NTS nephritis and harvested tissues 9 days after NTS induction (Figure 1A). Compared with MWT kidneys, we found that MKO kidneys exhibited significantly worsened glomerular injury as evidenced by higher glomerulosclerosis scores (Figure 1, B and C) and urinary albumin excretion (Figure 1D). In addition to glomerular pathology, compared with MWT kidneys, MKO kidneys displayed evidence of increased tubular injury as indicated by elevated *Havcr1*/kidney injury molecule 1 (KIM-1) and *Lcn2*/NGAL mRNA expression (Figure 1, E and F). Since we had previously demonstrated that loss of IL-1R1 in CD11c^+^ myeloid cells worsened ischemic AKI (14), we next tested whether deletion of IL-1R1 in CD11c^+^ myeloid cells exacerbated NTS nephritis. Mice with deletion of IL-1R1 in CD11c^+^ myeloid cells did not have worsened NTS nephritis compared with littermate mice (Supplemental Figure 2), suggesting the effect was likely mediated by a CD11c^lo^/CD11b^+^ myeloid cell. To further characterize the temporal progression of injury during NTS nephritis, we monitored urinary albumin excretion from day 6 to day 8 after NTS induction and found that compared with MWT mice, MKO mice exhibited higher albuminuria starting at day 7 (Supplemental Figure 3). As cross-model validation of susceptibility to chronic kidney damage in MKO mice, we confirmed that myeloid IL-1R1 deletion worsened kidney injury and fibrosis markers in a 28-day chronic ischemia/reperfusion (I/R) model, as evidenced by elevated KIM-1 and TGF-β expression and worsened histological injury scores in MKO kidneys compared with MWT controls (Supplemental Figure 4). Together, our data imply that LysM^+^ myeloid cell IL-1R1 deletion worsens disease progression in distinct chronic kidney injury models.

### LysM^+^ myeloid cell IL-1R1 deletion does not change IL-1Ra expression after NTS nephritis.

Previously, we demonstrated that F4/80^hi^ kidney macrophages produce IL-1Ra/*Il1rn* to limit inflammation 24 hours after septic kidney injury (39). We further showed a protective role for CD11c^+^ myeloid cell IL-1R1 to induce IL-1Ra generation and limit early ischemic kidney injury in a 24-hour I/R model (14). To determine whether reduced IL-1Ra expression could account for the worsened phenotype of MKO mice in the inflammatory NTS nephritis model, we measured *Il1rn* expression. At day 9 after NTS nephritis, myeloid IL-1R1 deletion did not differentially modulate *Il1rn* expression in the whole kidney (Supplemental Figure 5A), nor did it differentially affect numbers of infiltrating or resident CD11b^+^ kidney myeloid cells (Supplemental Figure 5, B–E). We further measured *Il1rn* expression in sorted CD11b^+^ kidney myeloid cells. Again, we did not find evidence that LysM^+^ myeloid cell IL-1R1 deletion changed *Il1rn* expression at day 9 after NTS nephritis (Supplemental Figure 5, F and G). Together, these results indicate that LysM^+^ myeloid cell deletion of IL-1R1 permits increased glomerular and tubular damage through a mechanism other than through regulation of IL-1Ra expression.

### The type I IFN response is important for NTS nephritis pathogenesis, and MKO mice display increased non-myeloid type I IFN expression.

To explore additional mechanisms important to the pathogenesis of NTS nephritis, we performed scRNA-seq on kidneys from WT mice either in control animals or at day 9 after induction of NTS nephritis. We clustered and annotated major kidney cell types by known genetic markers (41–43) (Supplemental Figure 6, A–C). We next performed pseudo-bulk analysis grouping all kidney cells by sample to identify global gene expression programs in the kidney after NTS nephritis. We performed differential expression analysis between control and day 9 NTS kidneys and found that of the 20 top differentially upregulated genes in NTS kidneys, 10 were IFN-stimulated genes (Figure 2, A and B). Moreover, many of these IFN-stimulated genes were also upregulated in kidney tubule, fibroblast, endothelial cell, and myeloid cell clusters (Supplemental Figure 6, D–G). Given the important role of type I IFN in autoimmune kidney diseases (27–30), we next examined whether MKO mice showed evidence of increased type I IFN signaling. Compared with kidneys from MWT mice, NTS-injured kidneys from MKO mice displayed increased nuclear levels of IFN regulatory factor 7 (IRF7) (Figure 2, C and D), a primary transcription factor involved in type I IFN expression (44), as well as modest but significantly increased *Ifnb1* mRNA expression in the whole kidney (Supplemental Figure 7). To determine whether MKO myeloid cells are the primary source of elevated type I IFNs in MKO-injured kidneys, we sorted CD11b^+^ myeloid cells from NTS-injured kidneys of MWT and MKO mice. Surprisingly, compared with MWT CD11b^+^ myeloid cells, we did not find evidence of increased type I IFN expression in MKO myeloid cells (Figure 2E). We next considered whether increased numbers of kidney myeloid cells in MKO kidneys might account for increased type I IFN expression; however, by flow cytometry, myeloid cell populations were similar in MWT and MKO kidneys (Figure 2, F–N). Taken together, these results imply that LysM^+^ myeloid cell IL-1R1 deletion increases type I IFN expression in the injured kidney, likely from a non-myeloid source.

### LysM^+^ myeloid cell IL-1R1 deletion increases type I IFN expression in endothelial cells after NTS nephritis.

To identify other potential sources of elevated type I IFNs after NTS nephritis, we performed gene ontology (GO) analysis of major kidney cell clusters from WT mice at baseline and day 9 after NTS induction (Figure 3A). Of kidney cell clusters, neutrophils, podocytes, and lymphocytes did not show upregulated GO pathways with a *q* value threshold less than 0.1. However, we did find upregulated GO pathways highly enriched in the type I IFN response (i.e., “response to virus,” “response to IFN-β”) across other kidney cell clusters including kidney tubules, fibroblasts, and endothelial cells (Figure 3, B–D). Moreover, we found that of kidney cell populations, endothelial cells, myeloid cells, neutrophils, and lymphocytes were the cell populations expressing high levels of *Irf7* after NTS nephritis (Figure 3E), indicating that type I IFNs could be generated from these cell types.

To ascertain the source of type I IFNs in MKO kidneys, we sorted kidney endothelial cells, fibroblasts, tubule cells, and myeloid cells from NTS-injured MWT and MKO mice (Figure 3F and Supplemental Figure 8A). Among these, only endothelial cells from MKO kidneys exhibited significantly elevated *Ifnb1* (Figure 3G) and *Ifna4* and *Ifna5* expression (Supplemental Figure 8, B and C), whereas type I IFNs were not differentially regulated by myeloid IL-1R1 expression in CD11b^+^ myeloid cells, fibroblasts, or tubular epithelial cells (Figure 3, H–J, and Supplemental Figure 8, B and C). These findings suggest that LysM^+^ myeloid cell IL-1R1 deletion selectively augments endothelial cell type I IFN expression after NTS nephritis.

### Ebi3/IL-27 increases endothelial cell type I IFN expression, and anti-IL27 ameliorates NTS nephritis in MKO mice.

To further evaluate crosstalk between kidney myeloid cells and endothelial cells after NTS nephritis, we performed NicheNet analysis (45) on our scRNA-seq dataset, setting the myeloid cell cluster as sender cells and endothelial cluster as receiving cells. We found that *Ebi3* is a highly prioritized ligand secreted from myeloid cells that can induce type I IFN–associated genes (i.e., *Irf7*, *Irf9*, *Ifit3*, *Stat1*) in endothelial cells (Figure 4A), and *Ebi3* is primarily expressed in myeloid cells (blue circle) in the kidney after NTS nephritis (Figure 4B).

EBI3 is one subunit of multiple heterodimeric cytokines: EBI3 combines with p28 (*Il27a*) to form IL-27; EBI3 combines with p35 (*Il12a*) to form IL-35; and EBI3 combines with IL-6 (*Il6*) to participate in IL-6 trans-signaling. To validate the potential EBI3/type I IFN axis, we analyzed expression of EBI3 binding partners *Il27a*, *Il12a*, and *Il6*. Among these, *Il27a* and *Il6*, but not *Il12a*, were significantly upregulated in MKO myeloid cells compared with MWT controls (Figure 4, C–F). To assess the potential of either IL-27 or IL-6 to regulate endothelial type I IFN expression, we cocultured b.End3 endothelial cells with CD11b^+^ myeloid cells sorted from NTS-injured kidney of MWT or MKO mice and in the presence or absence of neutralizing antibodies (Figure 4C). Endothelial *Ifnb1* expression was significantly higher after stimulation with MKO myeloid cells compared with MWT myeloid cells, and this increase was attenuated only by anti–IL-27 (Figure 4G) but not anti–IL-6 (Figure 4H). Given that NicheNet analysis also identified HMGB1 as a prioritized myeloid-derived ligand capable of targeting endothelial cells to induce type I IFN–associated genes (Supplemental Figure 9A), we additionally tested whether HMGB1 inhibition could attenuate the elevated endothelial type I IFN response. HMGB1 inhibition failed to ameliorate increased endothelial *Ifnb1* expression induced by MKO myeloid cells (Supplemental Figure 9B), suggesting that HMGB1 is not a primary mediator of this response. As further support of the detrimental effects of IL-27 during NTS nephritis in MKO mice in vivo, anti–IL-27 therapy was able to abrogate the difference in albuminuria and KIM-1 mRNA expression between MWT and MKO mice (Supplemental Figure 10).

Given that IL-27 has pleiotropic effects on CD4 Th cell differentiation, promoting Th1 and Treg responses and inhibiting Th2 and Th17 responses (18, 46–50), we next assessed T lymphocyte populations by flow cytometry in MWT and MKO mice after NTS nephritis. We found minimal differences between MWT and MKO mice in CD4 and CD8 populations and CD4 Th subsets (Supplemental Figure 11). There was actually a trend toward reduced IFN-γ^+^ CD4 T cells in MKO mice, which led us to believe the predominant effect of myeloid IL-1R1 deletion was not on lymphocytes.

In support of IL-27 modulating endothelial responses during NTS nephritis, in the scRNA-seq dataset, we found that endothelial cells express high levels of *Il27ra*, the receptor for IL-27. Moreover, subclustering of endothelial cells revealed that glomerular endothelial cells, a primary target of injury during NTS nephritis, expressed the highest relative levels of *Il27ra* (Figure 4, I–K); these results were supported in human CKD using the Kidney Precision Medicine Project (KPMP) tissue atlas (Supplemental Figure 12). Together, these findings identify a potential myeloid-to-endothelial crosstalk mechanism in which IL-1R1 suppresses *Ebi3*/IL-27 generation from myeloid cells, thereby limiting endothelial type I IFN expression after NTS nephritis.

### Protective effects of LysM^+^ myeloid cell IL-1R1 are predominantly from a non-neutrophil myeloid population.

Since LysM-Cre also mediates recombination in neutrophils (51), we next sought to determine whether neutrophils in MKO mice contributed to the dysregulated myeloid IL-27/endothelial type I IFN axis. We sorted neutrophils from MWT and MKO mice after NTS nephritis (Supplemental Figure 13A). In contrast with CD11b^+^Ly6G^–^ myeloid cells sorted from MKO kidneys, which showed elevated *Il27a* and *Il6* expression after NTS nephritis (Figure 4, D–F), Ly6G^+^ neutrophils isolated from MKO mice showed no difference in *Il27a* expression and significantly reduced *Il6* expression compared with MWT controls (Supplemental Figure 13, B and C). Moreover, compared with coculture with MWT neutrophils, coculture of MKO neutrophils with bEnd.3 endothelial cells actually reduced endothelial *Ifnb1* and *Ifna5* expression (Supplemental Figure 13, D–F; compare with Figure 4, G and H). To directly test the functional contribution of neutrophils in vivo, we depleted neutrophils using anti-Ly6G antibody during development of NTS nephritis (Supplemental Figure 13, G–I). Anti-Ly6G treatment was not able to ameliorate worsened glomerular and tubule injury in MKO mice (Supplemental Figure 13, J–L). Taken together, these findings suggest that other LysM^+^ myeloid cells, not neutrophils, are the primary contributor to increased endothelial type I IFNs and worsened NTS nephritis in MKO mice.

### Myeloid IL-1R1 deletion enhances ER stress–induced EBI3 expression.

We next wanted to further interrogate the potential mechanism whereby LysM^+^ myeloid IL-1R1 deficiency can lead to increased *Ebi3* expression. EBI3 is a resident ER protein whose expression can be increased by ER stress (52). To investigate whether IL-1R1 regulates the myeloid cell ER stress response, we sorted CD11b^+^ myeloid cells from NTS-injured kidneys and profiled genes associated with ER stress. Compared with MWT myeloid cells, MKO kidney myeloid cells exhibited higher expression of ER stress markers including PKR-like ER kinase (PERK; *Eif2ak3*), activating transcription factor 4 (ATF4; *Atf4*), binding immunoglobulin protein (BiP; *Hspa5*) and C/EBP-homologous protein (CHOP; *Ddit3*) (Figure 5, A and B). To determine whether IL-1R1 deficiency enhances ER stress in myeloid cells in vitro, we generated BM-derived macrophages (BMDMs) from MWT and MKO mice and stimulated them with the ER stress inducer tunicamycin for 6 hours (Figure 5C). Compared with MWT controls, MKO BMDMs showed significantly increased ER stress markers at both the mRNA (Figure 5, D–G) and protein levels (Figure 5, H–L). To assess whether these changes were also reflected at the whole kidney level, we measured ER stress marker expression in whole kidney lysates. In NTS-injured kidneys, MKO mice showed elevated expression of *Hspa5*, *Ddit3*, and *Eif2ak3* compared with MWT controls (Supplemental Figure 14A). Similarly, in the chronic I/R model, *Ddit3* expression was significantly increased in KO kidneys (Supplemental Figure 14B). Together, our data indicate that LysM^+^ myeloid cell IL-1R1 deletion increased ER stress both in vivo and in vitro.

We next asked whether ER stress regulates EBI3/IL-27 expression in myeloid cells in an IL-1R1–dependent manner. To this end, BMDMs from MWT and MKO mice were stimulated with the ER stress inducer tunicamycin for 6, 12, and 24 hours. Compared with MWT BMDMs, KO BMDMs displayed heightened expression of *Ebi3* and *IL27a* after ER stress induction (Figure 5, M–O). These results indicate that myeloid ER stress–induced EBI3/IL-27 expression is exaggerated in the absence of IL-1R1.

### Anti-IFNAR1 therapy ameliorates NTS nephritis in MKO mice.

Finally, to test whether LysM^+^ myeloid cell IL-1R1 limits autoimmune kidney injury by suppressing type I IFNs, we treated MWT and MKO mice with anti-IFNAR1 during NTS induction (Figure 6A). Anti-IFNAR1 treatment abrogated the differences in KIM-1 (Figure 6B), albuminuria (Figure 6C), and histological glomerular injury (Figure 6, D and E) between MWT and MKO mice. Taken together, these findings suggest that LysM^+^ myeloid IL-1R1 deletion worsens type I IFN–mediated NTS nephritis (Figure 6F).

## Discussion

In support of our hypothesis, we demonstrate here that myeloid IL-1R1 activity constrains glomerular and tubular injury in an autoimmune NTS nephritis model. Mechanistically, we found IL-1R1 mitigates ER stress in myeloid cells, inhibiting *Ebi3*/IL-27 expression and reducing endothelial type I IFN generation. Thus, our work lends further support to an important antiinflammatory and protective function of myeloid IL-1R1, here to constrain IL-27–mediated endothelial inflammation during autoimmune kidney damage.

In our study, endothelial type I IFNs played a major pathogenic role in NTS nephritis development. Indeed, the role of type I IFNs has been repeatedly linked to kidney damage during lupus (53), where the activation of type I IFNs is associated with kidney dysfunction (54). In addition, the administration of IFN-α and IFN-β impairs kidney function (55, 56); and interfering with signaling through their receptor, IFNAR, ameliorates these detrimental effects (57, 58). Moreover, type I IFNs induce apolipoprotein L1 (APOL1) expression in podocytes and endothelial cells and provide a second hit for development of APOL1 nephropathy in APOL1 G1 and G2 risk variant carriers (30, 59–62). Because myeloid cells are typically major producers of type I IFNs (63, 64), we initially thought that the increased type I IFNs we observed in KO mice would be due to heightened expression in myeloid cells after NTS nephritis. Surprisingly, we did not observe an increase in type I IFN expression in MKO myeloid cells relative to MWT myeloid cells after NTS, nor did we see increased numbers of myeloid cells in MKO kidneys. It is possible that we were unable to fully capture spatiotemporal differences in type I IFN generation by myeloid cells because our cell-sorting strategy to capture myeloid cells and measure type I IFNs was done at the whole-kidney level on day 9 at the end of the disease model. By contrast, we did find supportive evidence that the increased type I IFNs we observed in MKO kidneys after NTS emanated from endothelial cells. Although we cannot rule out the possibility that type I IFNs are also produced by other cell types, such as podocytes or lymphocytes, it should be noted that myeloid cells are distributed throughout the kidney in similar locations as endothelial cells. The complexity of these interactions between myeloid cells and other kidney parenchymal cell types, including endothelial cells, indicates that further studies are needed to fully elucidate myeloid cell–mediated cellular crosstalk and its effects on kidney disease.

Our study lends further support toward the very important role myeloid cells and endothelial cells play in regulating each other’s functions during kidney homeostasis and disease. Stamatiades et al. previously revealed that macrophages form an immunoregulatory axis with endothelial cells where they sample immune complexes in the glomerulus to activate the immune response (38). In a septic AKI model, we further demonstrated that cortical resident macrophages (glomeruli mainly reside in the cortex) limit septic AKI by inhibiting IL-6 generation from endothelial cells (39). Given that glomerular endothelial cells are the primary initial site of injury during immune complex deposition in the kidney, it is plausible that a primary mechanism through which activated myeloid cells signal to glomerular endothelial cells is through IL-27. Indeed, we found that of kidney endothelial subtypes, glomerular endothelial cells are the primary subtype that expresses the receptor for IL-27, *Il27ra/IL27RA*, in both mice and humans. Whether this IL-27–mediated crosstalk between myeloid cells and endothelial cells is a common inciting mechanism of immune complex–mediated damage in the kidney remains to be determined.

Regarding IL-27, while both pro- and antiinflammatory roles have been ascribed to IL-27, our study provides support for a proinflammatory and likely detrimental role in endothelial cells. After induction, IL-27 subunit EBI3 is retained in the ER, where it can bind p28. EBI3 is a chaperone to ensure correct protein folding of p28 to form IL-27 (65). In the kidney, our data reveal that myeloid cells are major expressers of *Ebi3*. After release, IL-27 binds to its receptor, the IL-27 receptor, which is a heterodimer of the IL-27 receptor (WSX-1/*Il27ra*) and glycoprotein 130 (gp130). Downstream signaling subsequently occurs through JAK/STAT — mainly STAT1 and STAT3, p38 and ERK MAPK, and NF-κB/activator protein-1 pathways (16). Besides endothelial cells, we found that the main cell type expressing *Il27ra* is lymphocytes. Indeed, the role of IL-27 in T cell differentiation has received considerable attention as it induces Th1 differentiation, inhibits Th2 and Th17 responses, and enhances the cytotoxic function of CD8^+^ T cells (17, 18). We were unable to identify significant upregulation of GO pathways associated with type I IFNs in kidney lymphocytes from our scRNA-seq dataset. We also did not observe differences in numbers of CD4 Th lymphocyte subsets between MWT and MKO mice by flow cytometry after NTS nephritis. However, we did find that anti–IL-27 was able to abrogate type I IFN generation from endothelial cells cocultured with MKO myeloid cells in vitro and reverse NTS nephritis in MKO mice in vivo. Our data are consistent with a previously demonstrated role for IL-27 to induce type I IFN–associated genes (19–23) and proinflammatory activation of endothelial cells (66–68). Thus, modulating IL-27 signaling in endothelial cells holds considerable promise as a therapy for kidney autoimmune diseases where endothelial damage plays a critical role.

In our study, myeloid IL-1R1 deficiency was associated with increased *Ebi3*/IL-27 expression. Our findings add yet another layer of complexity to the complicated role of IL-1/IL-1R1 in kidney disease. Most early studies using global IL-1R1–deficient animals revealed that activation of IL-1R1 leads to worse AKI and/or fibrosis after I/R (7), unilateral ureteral obstruction (8), cyst formation (10), and cisplatin-induced AKI (11). Studies using mice with cell-specific deletion of IL-1R1 in kidney tubule epithelium further revealed a detrimental role for IL-1R1 in kidney disease (9, 12). In contrast, IL-1R1 on podocytes (15) and endothelial cells (12) limits toxic AKI. Moreover, our recent study using mice with cell-specific deletion of IL-1R1 in CD11c^+^ myeloid cells revealed that IL-1R1 serves as a negative feedback regulator at 24 hours after ischemic kidney injury by promoting release of the antiinflammatory cytokine IL-1Ra (14). In this study, we did not find similar effects of myeloid IL-1R1 to regulate IL-1Ra expression after NTS nephritis, though it should be noted that our acute I/R model is characterized by a rapid, innate immune response in which IL-1Ra may provide an early negative feedback mechanism. In contrast, the antibody-mediated NTS nephritis model reflects a more sustained inflammatory process. Nevertheless, we did find that IL-1R1 constrains myeloid ER stress and subsequent *Ebi3* expression. Cellular stress can disrupt normal protein folding and cause accumulation of abnormal proteins in the ER, which leads to ER stress and the unfolded protein response (69). Unmitigated ER stress has been implicated in multiple kidney diseases including AKI, glomerulonephritis, and kidney fibrosis (69–74). In macrophages, chronic ER stress alters cytokine production and promotes inflammation. ER stress can also induce Ebi3 expression (52). Thus, a primary mechanism through which IL-1R1 serves as a negative feedback regulator of myeloid cell activation could be through reduction of pathways leading to ER stress. These studies remain an active area of investigation.

Several limitations of this study warrant consideration. First, LysM-Cre targets a broad myeloid compartment rather than macrophages exclusively. Our neutrophil depletion and CD11c-Cre experiments suggest that neither neutrophils nor CD11c^hi^/CD11b^–^ cells are the primary drivers of the observed phenotype. Our findings are therefore best interpreted within the context of a non-neutrophil CD11b^+^/LysM^+^ myeloid compartment that includes monocytes and macrophages. Second, certain gene expression changes were detectable in sorted cell populations but not at the whole kidney level, which may reflect cellular heterogeneity, potential compensatory mechanisms within the kidney, or spatially localized expression differences that cannot be resolved at the whole kidney level. Third, although our IL-27 neutralization experiments provide strong support for myeloid-derived IL-27 as a key driver of endothelial type I IFN responses in MKO mice, additional mechanisms may also contribute to the broader kidney injury phenotype in this model. Last, the NTS nephritis model is a classic model of immune-mediated glomerulonephritis. Thus, the effects of myeloid IL-1R1 to influence endothelial type I IFN expression should be interpreted within this disease context. Nevertheless, our data from both the NTS nephritis and chronic I/R models reveal that myeloid IL-1R1 might reduce ER stress, the mechanism of which is an area of ongoing investigation.

In conclusion, IL-1R1 on myeloid cells plays a crucial role in constraining autoimmune glomerular and tubular injury by inhibiting ER stress–mediated IL-27 generation, which in turn alleviates type I IFN expression from kidney endothelial cells. These findings provide a compelling rationale to target this interaction as a therapeutic approach in autoimmune kidney injury and potentially other autoimmune diseases where myeloid cells and endothelial cells play a role in pathogenesis.

## Methods

### Sex as a biological variable.

Our study examined male and female animals, and similar findings are reported for both sexes. In all figures, female animals are represented by circular symbols and male animals by square symbols.

### Animal care.

The 129/SvEv male *IL-1R1^fl/fl^* mice (75), a gift from Matthew Robson (University of Cincinnati College of Medicine, Cincinnati, Ohio, USA) and Randy Blakely (Florida Atlantic University, Boca Raton, Florida, USA), were bred with 129/SvEv female *LysMCre*^+^ mice (51) to generate *LysMCre*^+^*/Il1r1^fl/fl^* (MKO) mice and *LysMCre^–^/Il1r1^fl/fl^* (MWT) littermates. C57BL/6J male *IL-1R1^fl/fl^* mice were bred with female *CD11cCre*^+^ mice (The Jackson Laboratory, strain 008068, C57BL/6J congenic background) to obtain *CD11cCre*^+^*/IL-1R1^fl/fl^* mice and *CD11cCre^–^/IL-1R1^fl/fl^* littermate control mice. All mice were backcrossed at least 8 times onto the parent genetic strain.

### NTS nephritis model.

Male or female mice 12–16 weeks old were randomly assigned to the baseline or NTS nephritis model based on cage. Mice were injected intraperitoneally with 250 μg of sheep IgG (I5131, Sigma-Aldrich) in 100 μL of sterile PBS containing Freund’s adjuvant (F5881, Sigma-Aldrich) on day 0. On day 5, mice received a tail vein injection of sheep anti-mouse GBM serum (PTX-001S, Probetex). On day 9, mice were harvested for urine, blood, and kidney tissue. The primary outcomes for NTS were albuminuria and kidney mRNA levels of NGAL/*Lcn2* and KIM-1 (*Havcr1*). Baseline MWT mice were used as the control group. No a priori exclusion criteria were utilized, and all mice were analyzed. Experimenters were blinded to genotype and treatment until primary outcome measurements were obtained. Body weight was monitored and did not differ between groups.

### Chronic I/R injury model.

Chronic kidney injury was induced using a unilateral I/R model with delayed contralateral nephrectomy (76). Briefly, on day 0, mice were anesthetized with ketamine (100 mg/kg) and xylazine (10 mg/kg) and underwent left renal pedicle clamping for 38 minutes with an 800 g pressure clamp (FST 18052-03), followed by visual confirmation of reperfusion upon clamp removal. Mice were maintained on a heating pad at 38°C during the procedure to preserve body temperature. On day 8, the contralateral (right) kidney was surgically removed under isoflurane anesthesia (3%) via nose cone. Mice were then monitored and followed until day 28, at which time samples were collected for downstream analyses. Sham-operated controls underwent identical surgical procedures without vascular clamping.

### Anti-IFNAR1 treatment.

MWT and MKO mice were randomly assigned to receive intraperitoneal injections of either vehicle or anti–IFNAR1 antibody (BE0241, Bio X Cell, RRID AB_2687723) at day 5 (500 μg/mouse), day 6 (500 μg/mouse), and day 8 (250 μg/mouse) of the NTS experiment (77). Mice were euthanized on day 9.

### Anti–IL-27p28 treatment.

MWT and MKO mice were randomly assigned to receive intraperitoneal injections of either vehicle or anti–IL-27p28 antibody (BE0326, Bio X Cell, RRID AB_2819053) at day 5 (500 μg/mouse), day 6 (500 μg/mouse), day 7 (500 μg/mouse), and day 8 (250 μg/mouse) of the NTS experiment (78). Mice were euthanized on day 9.

### Anti-Ly6G treatment.

MWT and MKO mice received intraperitoneal injections of anti-Ly6G antibody (BE0075-1, Bio X Cell, RRID AB_1107721) at day 5 (200 μg/mouse), day 7 (200 μg/mouse), and day 8 (200 μg/mouse) of the NTS experiment (35). Mice were euthanized on day 9.

### Urinary albumin measurement.

Urine was collected directly from the bladder and centrifuged at 13,800*g* rpm for 10 minutes at 4°C. Urine albumin and creatinine levels were measured with kits (Ethos Biosciences, 1011 and 1012, respectively) according to the manufacturer’s instructions. Urinary albumin excretion was expressed as albumin/creatinine ratio.

### Histological injury scoring.

Kidney tissues were removed and a cross-sectional segment obtained. The kidney segment was fixed with 10% neutral-buffered formalin (VWR 16004-128), embedded with paraffin, and sectioned in 5 μm sections by the Duke Research Immunohistology Laboratory. Sections were stained with periodic acid–Schiff (PAS) stain and scored by an experienced animal pathologist masked to experimental groups. For histological injury scores, sections were graded according to a previously established scoring system (9): the percentages of tubules with cell lysis/dilation, loss of brush border, and cast formation were scored on a scale from 0 to 4 (0, no damage; 1, less than 25%; 2, 25%–50%; 3, 50%–75%; 4, >75%). Histological scores for each kidney were obtained by adding individual component scores. For glomerulosclerosis score, an investigator who was blinded to the experimental conditions scored at least 20 random glomeruli under the microscope. A score of 0 denotes that the percentage of sclerotic glomeruli was less than 5%, and scores of 1, 2, 3, 4, and 5 denote 6%–20%, 21%–40%, 41%–60%, 61%–80%, and greater than 81% of the selected glomeruli, respectively. A score was assigned to each mouse.

### Immunohistological staining.

Kidney segments were fixed in 10% neutral-buffered formalin, paraffin-embedded, and sectioned at 5 μm by the Duke Research Immunohistology Laboratory. For IHC, paraffin sections were deparaffinized and rehydrated before antigen retrieval in citrate solution (14746, Cell Signaling Technology) by microwave heating. Endogenous peroxidase activity was blocked with 3% hydrogen peroxide for 10 minutes, followed by blocking in 1% BSA for 1 hour at room temperature. Sections were then incubated with anti-Ly6G antibody (87048, Cell Signaling Technology) overnight at 4°C, washed, and incubated with HRP-conjugated secondary antibody (8114, Cell Signaling Technology) for 1 hour at room temperature. Signal was developed using DAB substrate (8059, Cell Signaling Technology) for 5 minutes, and sections were counterstained with hematoxylin (H-3401, Vector Laboratories) for 2 minutes before dehydration and coverslip mounting. Images were acquired on an Axio microscope (ZEISS). Ly6G^+^ neutrophils were manually enumerated across the entire kidney section using ImageJ (NIH) per section. All analyses were carried out by researchers blinded to experimental group assignments.

### RNA extraction and real-time quantitative PCR.

RNA was isolated with a Quick-RNA MiniPrep (R1055, Zymo Research) or Quick-RNA MicroPrep (R1501, Zymo Research) according to the kit instructions. RNA concentration was measured by Nanodrop. The High-Capacity cDNA Reverse Transcription kit (4368813, Invitrogen) was used to synthesize cDNA according to the manufacturer’s instructions. Gene expression levels were measured by RT-PCR using a TaqMan assay (4370074, Applied Biosystems) and primers (Supplemental Table 1).

### Nuclear and cytoplasmic protein extraction.

Kidney tissues were weighed and extracted for nuclear and cytoplasmic protein according to the kit manufacturer’s instructions (78835, Thermo Fisher Scientific). Protein concentrations were measured using Pierce BCA Protein assay kit (23227, Thermo Fisher Scientific). Protein for each sample was denatured in boiling water for 10 minutes.

### Western blotting.

Equal amounts of total protein from the samples were loaded into 4%–12% bis-tris gels and transferred to PVDF membranes. The membranes were blocked by 5% nonfat milk-TBST for 1 hour and then incubated with primary antibodies overnight at 4°C. After washing and incubation with secondary antibodies, membranes were then processed with chemiluminescence (WBLUC0500, MilliporeSigma) and detected with an Amersham gel imager 680 analysis system. The detected bands were quantified for densitometry by ImageJ (NIH). Primary antibodies included anti-IRF7 (39659, Cell Signaling Technology, RRID:AB_2942011), anti-histone-3 (9715, Cell Signaling Technology, RRID:AB_331563), anti-CHOP (2895, Cell Signaling Technology, RRID:AB_2089254), anti-BiP (3177, Cell Signaling Technology, RRID:AB_2119845), anti-ATF4 (11815, Cell Signaling Technology, RRID:AB_2616025), anti-phosphorylated PERK (MA5-15033, Thermo Fisher Scientific, RRID:AB_10980432), and anti-β-actin (A1978, Sigma-Aldrich, RRID:AB_476692).

### Kidney flow cytometry and cell sorting.

Kidneys were removed from NTS-injured MWT and MKO mice and minced into small pieces, and then transferred into a GentleMACS C tube containing 5 mL of digestion buffer (RPMI-1640, 10 μg/mL DNase, 1 mg/mL type IV collagenase). The minced kidney was processed using GentleMACS Dissociator. After mechanical dissociation, the kidney mixture was digested for 30 minutes at 37°C. Cells were soft spun down at 10 *g* for 1 minute, and supernatant was filtered through a 70 μm cell strainer to remove large cell debris and washed with wash buffer (Dulbecco’s PBS, 3% FBS, 2 mM EDTA). Cells were spun down, and red blood cells in pellets were lysed with ACK lysis buffer (A10492-01, Gibco), and then the cell suspensions were filtered through a 40 μm cell strainer. Cells were spun down, resuspended with Fc-block buffer (101320, BioLegend), and incubated for 15 minutes at 4°C before staining with fluorescently labeled antibodies (Supplemental Table 2), and then live/dead near-IR Dead Cell stain (L34976, Invitrogen). Cells were fixed with CytoFix (554655, BD Biosciences). Prior to analysis, 20 μL of Count Bright absolute counting beads (C36950, Invitrogen) were added to cells, and samples were acquired on a BD Biosciences LSRII and analyzed using FlowJo software. Total cell numbers were obtained using an enumeration formula as described in the manufacturer’s instructions. For cell sorting experiments, at the end of primary antibody incubation, cells were spun down and resuspended in Dulbecco’s PBS containing 2% FBS, 2 mM EDTA, 10 μg/mL DNase, and DAPI (1:10,000), and live cells were sorted for populations of interest on an Astrios Sorter (Beckman) by the Duke Cancer Institute Flow Cytometry Core. Cells were collected directly into RNA lysis buffer and used for gene expression analysis.

### Intracellular cytokine staining.

After surface marker staining and viability dye labeling, cells were fixed and permeabilized using the Foxp3/Transcription Factor Staining buffer set (00-5523-00, Thermo Fisher Scientific) according to the manufacturer’s instructions. Briefly, cells were incubated in fixation/permeabilization buffer for 60 minutes, washed with permeabilization buffer, and blocked with 2% normal rat serum (31888, Thermo Fisher Scientific). Cells were then stained with fluorochrome-conjugated antibodies against intracellular cytokines (Supplemental Table 2) in permeabilization buffer for 30 minutes at room temperature protected from light. After washing, cells were resuspended in flow cytometry staining buffer and acquired on a BD Biosciences LSRII flow cytometer. Data were analyzed using FlowJo software.

### BMDM culture.

BMDMs were prepared as described (79, 80). Briefly, BM cells were harvested from MWT or MKO mice by flushing the femurs and tibia with 1× Dulbecco’s PBS containing 2% FBS and 2 mM EDTA. Cells were then resuspended and cultured in RPMI media (20% heated-inactivated FBS with 20 mM penicillin/streptomycin) supplemented with M-CSF (20 ng/mL, 576406, BioLegend) to differentiate them into macrophages, which were verified by CD11b and F4/80 staining by flow cytometry. After 7–10 days induction, the mature BMDMs were utilized for experiments. Mature BMDMs derived from MWT and MKO mice were stimulated with either vehicle or tunicamycin (1 μg/mL, T7765, Sigma-Aldrich) for 6 hours. After the stimulation, both the cell supernatants and cell lysates were harvested for further analysis.

### bEnd.3.cell culture.

The mouse immortalized endothelial cells (CRL-2299, ATCC) were purchased from Duke Cell Culture Facility and DNA Lab and cultured in DMEM supplemented with 10% FBS and 1% penicillin/streptomycin. bEnd.3 cells were cultured in 12-well plates to 80%–90% confluence. The cells were then cocultured with CD11b^+^ cells sorted from MWT and MKO NTS nephritis kidneys for 12 hours in the presence or absence of anti-IL27p28 (25 μg/mL; BE0326, Bio X Cell, RRID AB_2819053) or anti-IL6 (25 μg/mL; BE0046, Bio X Cell, RRID AB_1107709) or HMGB1 inhibitor (250 μM, Glycyrrhizin, NSC 167409, Selleckchem). Subsequently, the bEnd.3 cells were harvested for RNA analysis.

### scRNA-seq analysis.

Our scRNA-seq cell isolation protocol has been previously described (39, 81). Mice were perfused with ice-cold PBS via cardiac puncture, after which whole kidneys were harvested. Kidney digest was performed with Liberase (0.3 mg/mL, 291963, Roche), hyaluronidase (10 mg/mL, H4272, Sigma-Aldrich), and DNase I (20 mg/mL) at 37°C for 20 minutes. After centrifugation, cell pellets were incubated with ACK lysing buffer (A1049201, Thermo Fisher Scientific) and again centrifuged. Pellets were incubated with 0.25% trypsin EDTA at 37°C for 10 minutes with shaking at 100 revolutions per minute in a bacterial shaker. Trypsin was inactivated using 10% FBS in PBS. Cells were then washed and resuspended in Dulbecco’s PBS supplemented with 0.04% BSA. After filtration through a 30 mm strainer, and after viability was determined to be greater than 90%, cells were loaded onto a 10X Chromium System (10X Genomics) at 1,000 cells/mL by the Duke Human Vaccine Institute Sequencing Core. The vehicle control condition consisted of pools of kidney digests from 3 WT mice treated with saline vehicle; the day 9 condition consisted of a pool of kidney digests from 3 WT mice treated with NTS protocol and harvested at day 9. After library preparation, samples were sequenced on a NovaSeq 6000 (Illumina) S2 flow cell by the Duke Center for Genomic and Computational Biology Sequencing Core. Cell Ranger software from 10X Genomics was used to transform raw base call files into FASTQ files (cellranger mkfastq) and align reads with reference mouse genome (cellranger count).

For quality control and filtering, gene expression matrices generated separately by the *CellRanger* pipeline for each of the 2 samples were merged together into a single matrix. To exclude low-quality cells, we filtered out cells for which fewer than 200 genes/500 unique molecular identifiers (UMIs) were detected and excluded cells with greater than 9,000 genes/150,000 UMIs. We also filtered out cells for which the percentage of mitochondrial gene expression represented more than 60% of the total gene expression to avoid excluding mitochondrial-rich proximal tubule cells (42). These filters removed 1,990 cells. The dataset ultimately consisted of 14,684 cells (6,340 for vehicle; 8,344 for day 9 NTS). All genes that were not expressed in at least 1 cell were discarded, leaving 23,113 genes.

For normalization, sample integration, and clustering, we used the R (v4.3.2) package Seurat (v5.2.1) standard workflow for integration of multiple samples to combine the 2 samples into a unique dataset before clustering and visualization. Prior to integration, gene expression was normalized for each sample by scaling by the total number of transcripts, multiplying by 10,000, and then log-transforming (log-normalization). We then identified the 2,000 genes that were most variable across each sample. Next, we identified anchor genes between samples using the *FindIntegrationAnchors* function that were used as a basis for Seurat to perform the integration of the 2 samples. We scaled the integrated data before running a principal component analysis (PCA). We then utilized the shared nearest neighbor (SNN) modularity optimization-based clustering algorithm implemented in Seurat for identifying clusters of cells. This was performed using the *FindNeighbors* function with 30 principal components, followed by the *FindClusters* function with the Louvain algorithm using a 0.2 resolution. This allowed us to assign cells into a total of 16 clusters. We applied the UMAP method on the cell loadings of the previously selected 30 principal components to visualize the cells in 2 dimensions. Known markers of cells present in the mouse kidneys were used to manually identify corresponding cell types for each cluster (41–43). After annotation and combining of cell subpopulations (i.e., all tubule segments together), the final clustering consisted of 7 different cell populations. Expression of genes of interest were plotted using the DotPlot and FeaturePlot functions within Seurat on the RNA assay.

Differential expression analyses and GO enrichment analysis were performed using the function *FindMarkers* implemented in the Seurat package with default parameters and Wilcoxon’s rank-sum test (day 9 NTS vs. vehicle). Differentially expressed genes were selected with the following threshold: adjusted *P* value less than 0.05. GO enrichment analysis was performed in R for upregulated biological processes (82) using the enrichGO function in package clusterProfiler using a *P* value cutoff of 0.05, *q* value cutoff of 0.1, and log_2_ fold-change greater than 0.5.

For subclustering of endothelial cells, endothelial cells were subsetted from an integrated Seurat object. We then identified the 2,000 genes that were most variable across each sample. Next, we identified anchor genes between samples using the *FindIntegrationAnchors* function that were used as a basis for Seurat to perform the integration of the 2 samples. We scaled the integrated data before running a PCA. We then utilized the SNN modularity optimization-based clustering algorithm implemented in Seurat for identifying clusters of cells. This was performed using the *FindNeighbors* function with 30 principal components, followed by the *FindClusters* function with the Louvain algorithm using a 0.1 resolution. This allowed us to assign cells into a total of 4 clusters. We applied the UMAP method on the cell loadings of the previously selected 30 principal components to visualize the cells in 2 dimensions. Known markers of cells present in the mouse kidneys were used to manually identify corresponding cell types for each cluster (41, 83). After annotation and combining of cell subpopulations, the final clustering consisted of 3 clusters based on naming by Dumas et al. (83).

The NicheNet package was used to identify potential ligands/receptors/target gene interactions between endothelial cells and myeloid cells with default parameters (45). We specifically identified the myeloid cell cluster as sender cells and endothelial cells as receiver cells.

### Statistics.

Figures where indicated were generated using BioRender.com (https://BioRender.com/q8i90x6). Statistical analysis for scRNA-seq analysis was performed as described in *scRNA-seq analysis*. Statistical tests and visualization for all other data were performed with GraphPad Prism (v10.2.1). As indicated, the data are visualized in dot plots (for each sample) with the line at median or box plots with the box representing the 25th–75th percentile, line at median, and whiskers extending min to max. The figures are representative of experiments that were repeated at least twice on different days. An unpaired 2-tailed Student’s *t* test with Welch’s correction was used to compare 2 experimental groups with normal distribution, a Mann-Whitney *U* test was used to compare 2 experimental groups with ordinal data. Comparisons involving more than 2 groups were analyzed by 2-way ANOVA with Šídák’s multiple-comparison test for post hoc analysis or multiple-comparison *t* test as appropriate to compare groups. *P* less than 0.05 was considered statistically significant.

### Study approval.

All of the animal studies were approved by the Durham Veterans Affairs Medical Center (VAMC) IACUC, performed at the Durham VAMC, and conducted in accordance with the NIH *Guide for the Care and Use of Laboratory Animals* (National Academies Press, 2011).

### Data availability.

All data reported in this paper are available in the Supporting Data Values file. scRNA-seq data have been deposited in NCBI’s Gene Expression Omnibus (GEO GSE274248) and are publicly available as of the date of publication. All original code has been deposited at Duke Gitlab at https://gitlab.oit.duke.edu/jrp43/privratskylab_code.git and is publicly available as of the date of publication.

### Prior publication.

This work was presented as an accepted abstract at the American Society of Nephrology Kidney Week 2024 annual meeting in San Diego, California, USA, October 23–27, 2024.

## Author contributions

YC, JR, SDC, and JRP were involved in designing research studies. YC, YL, JR, CCW, XL, AI, and JRP were involved in conducting experiments. YC, YL, JR, CCW, XL, AI, and JRP were involved in acquiring data. YC, JR, SDC, JRP were involved in analyzing data. SDC and JRP provided reagents. YC, YL, JR, CCW, XL, AI, SDC, and JRP were involved in writing the manuscript.

## Conflict of interest

The authors have declared that no conflict of interest exists.

## Funding support

This work is the result of NIH funding, in whole or in part, and is subject to the NIH Public Access Policy. Through acceptance of this federal funding, the NIH has been given a right to make the work publicly available in PubMed Central.

- NIH grant K08GM132689 (to JRP).
- NIH grant R01DK131065 (to JRP).
- NIH grant R01DK118019 (to SDC).
- US Veterans Health Administration, Office of Research and Development, Biomedical Laboratory Research and Development grant BX000893 (to SDC).
- Laboratory Research and Development grant BX000893 (to SDC).
- VA Clinician Scientist Investigator Award (to SDC).

## Supplementary Material

Supplemental data

Unedited blot and gel images

Supporting data values

## Figures and Tables

**Figure 1:**
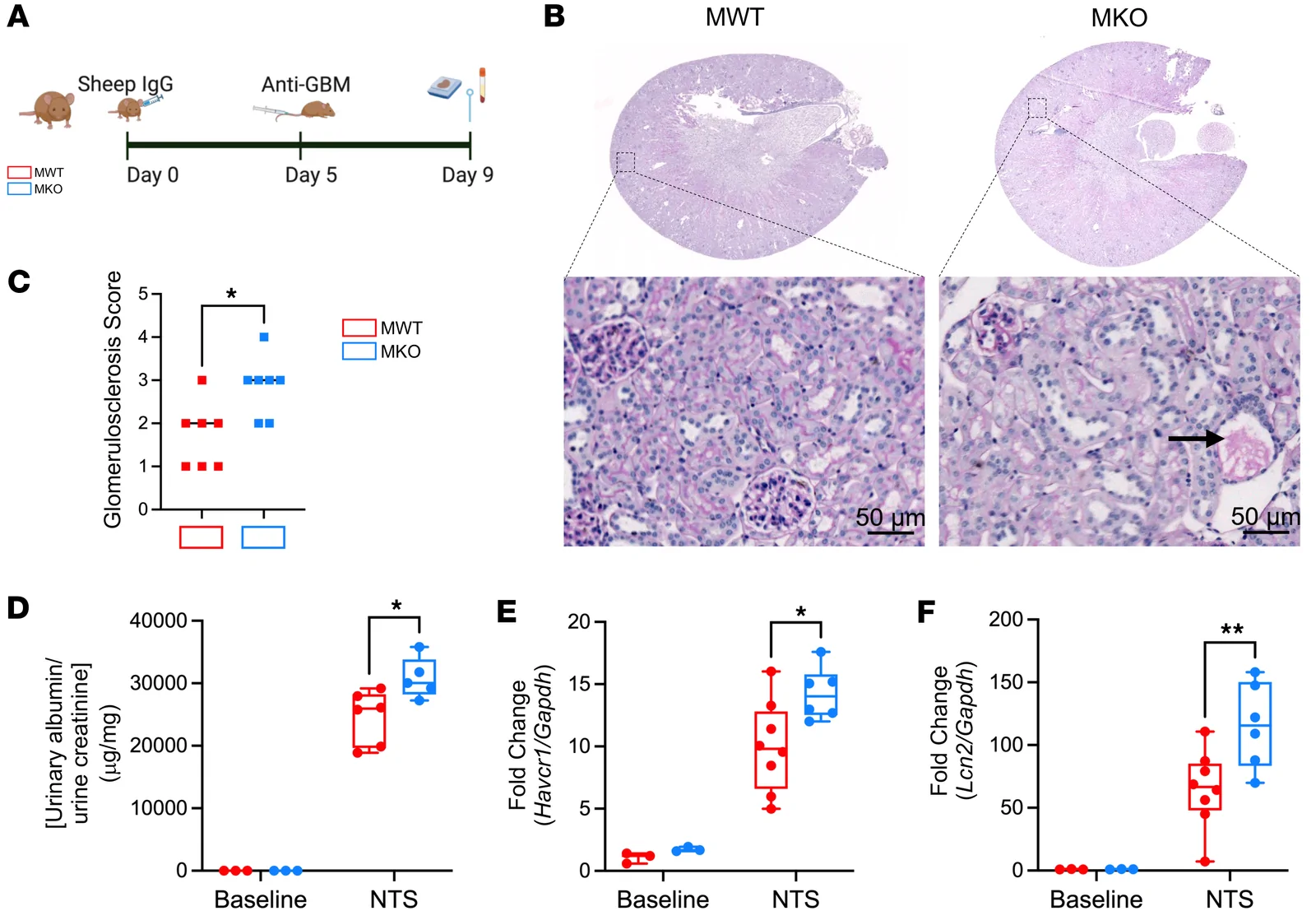
Myeloid IL-1R1 deletion worsens NTS nephritis. (**A**) MWT and MKO mice were subjected to NTS nephritis, and tissue was harvested at 9 days as shown in the schematic (created with BioRender.com). (**B**) Representative 40× PAS images from kidney sections (arrowhead represents collapsed glomerulus; scale bar: 50 μm), with (**C**) glomerulus injury score assessed with a blinded protocol (*n* = 7 per group; squares indicate individual animals with line at median, *P < 0.05 by Mann-Whitney *U* test). (**D**–**F**) Levels of (**D**) urinary albumin were measured and normalized to urinary creatinine (*n* = 3 for baseline groups; *n* = 5–6 for NTS groups; data represented in box plots, *P < 0.05 by 2-way ANOVA with Bonferroni’s post hoc test). mRNA levels of kidney injury markers (**E**) KIM-1 (*Havcr1*) and (**F**) NGAL/*Lcn2*. Graphs display box plots with dots indicating individual samples (*n* = 3 for baseline groups; *n* = 6–8 for NTS groups; *P < 0.05, **P < 0.01 by 2-way ANOVA with Bonferroni’s multiple-comparison test. Note: sample size differences between urine albumin and KIM-1 and NGAL measurements are due to inability to collect urine in some mice. Circles represent female animals and squares represent male animals.

**Figure 2:**
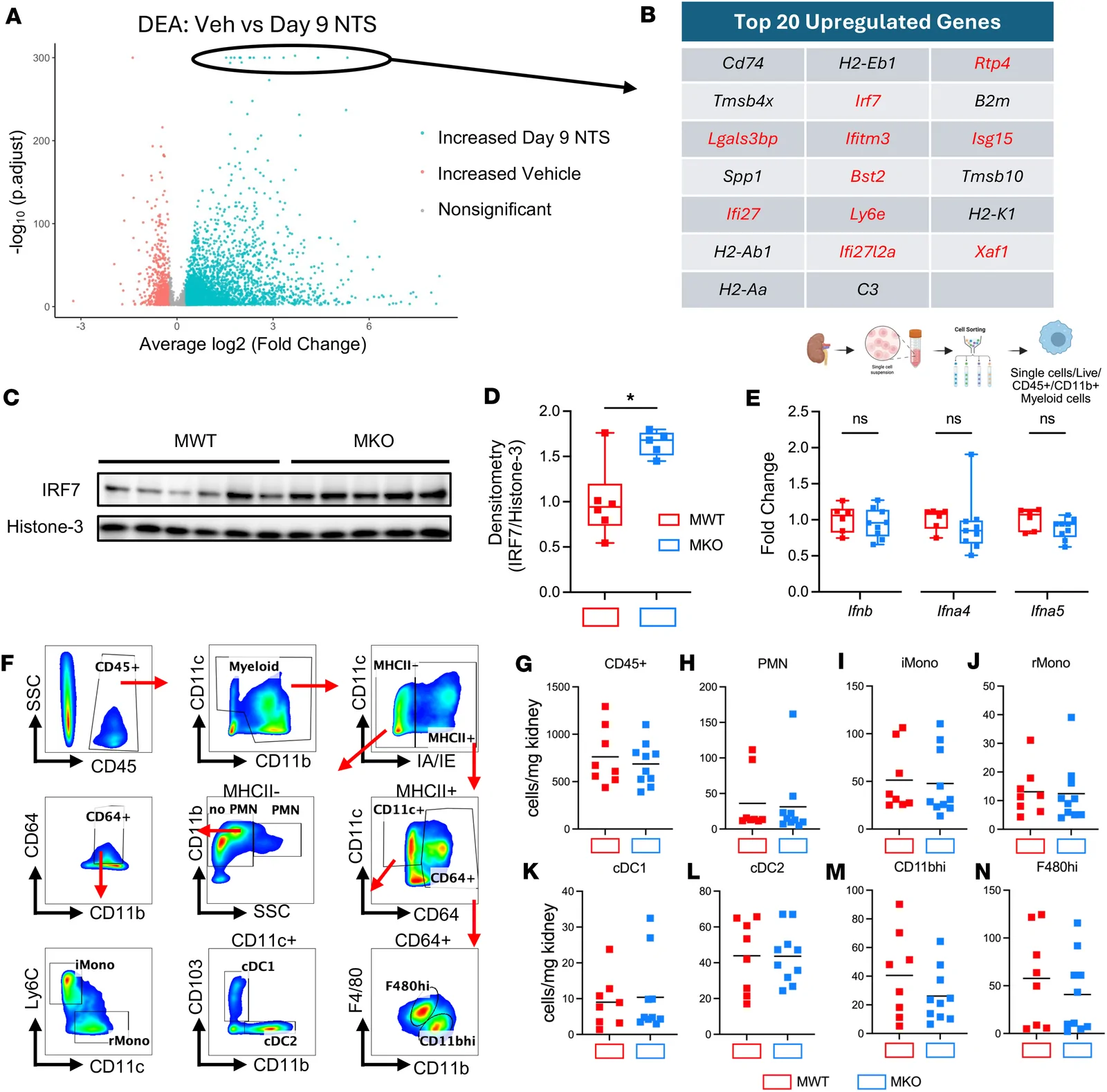
NTS nephritis induces IFN-stimulated genes, and MKO mice display elevated type I IFN signaling. (**A** and **B**) Kidneys from vehicle (Veh) or NTS nephritis–injured mice (day 9) were harvested, scRNA-seq was performed on whole kidney digests, and pseudo-bulk analysis was performed grouping all kidney clusters together by sample (Veh or day 9). (**A**) Volcano plot showing differentially expressed genes between Veh and day 9 kidneys with (**B**) top 20 significantly upregulated genes in day 9 NTS kidneys (IFN-stimulated genes in red). (**C**–**E**) MWT and MKO mice were subjected to NTS nephritis, and kidneys were harvested at day 9 with Western blot (**C**) and densitometry (**D**) for nuclear levels of IRF7 displayed in box plot (*n* = 5–6; *P < 0.05 by unpaired 2-tailed *t* test). (**E**) CD11b^+^ myeloid cells (live/single cell/CD45^+^Ly6G^–^CD11b^+^) were sorted from NTS nephritis–injured MWT and MKO kidneys, RT-PCR was performed, and box plots show mRNA levels of *Ifnb1*, *Ifna4*, *Ifna5* (*n* = 6–9; not significant by unpaired 2-tailed *t* test; schematic created with BioRender.com). (**F**–**N**) Flow cytometry was performed on NTS nephritis injured–MWT and MKO whole kidney digests with gating strategy for cell populations shown in pseudocolor plots in **F**. Dot plots display enumeration for (**G**) CD45^+^ (live/single cell), (**H**) neutrophils (PMN, CD45^+^CD11b^+^Ly6G^+^), (**I**) Ly6c^hi^ monocytes (iMono, CD45^+^Ly6G^–^MHCII^–^CD64^+^Ly6C^hi^), (**J**) Ly6c^lo^ monocytes (rMono, CD45^+^Ly6G^–^MHCII^–^CD64^+^Ly6C^lo^), (**K**) conventional DC 1 (cDC1) (CD45^+^Ly6G^–^MHCII^+^CD64^–^CD11c^+^CD103^hi^CD11b^–^), (**L**) cDC2 (CD45^+^Ly6G^–^MHCII^+^CD64^–^CD11c^+^CD103^lo^CD11b^+^), (**M**) CD11b^hi^ macrophages (CD11b^hi^, CD45^+^Ly6G^–^MHCII^+^CD64^+^CD11b^hi^F4/80^lo^), and (**N**) F480^hi^ macrophages (F480^hi^, CD45^+^Ly6G^–^MHCII^+^CD64^+^CD11b^mid^F480^hi^). There were no statistically significant differences between genotypes for any cell population as determined by unpaired 2-tailed *t* tests (*n* = 8–10). Squares represent male animals.

**Figure 3:**
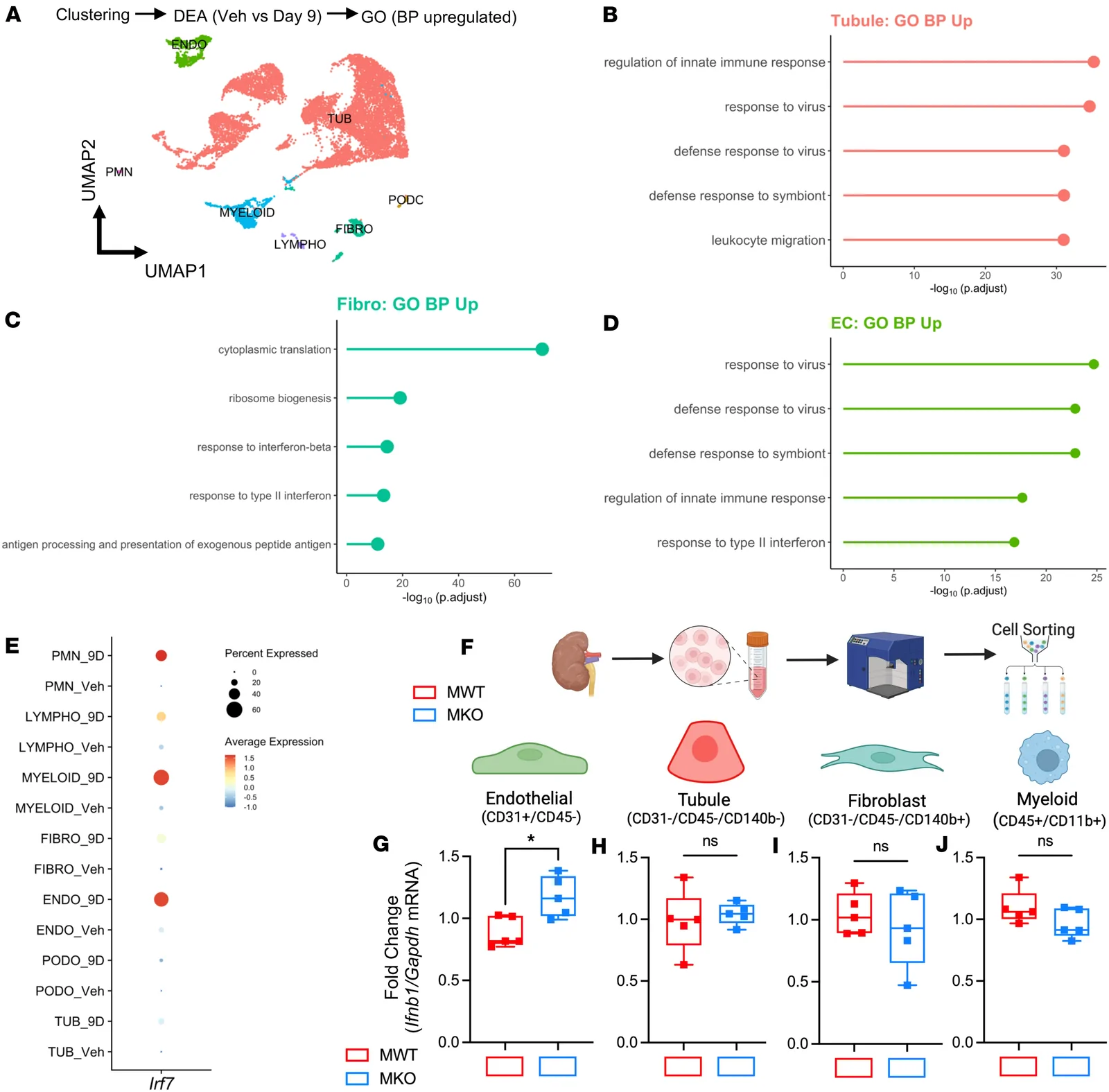
MKO mice have increased endothelial type I IFN generation after NTS nephritis. (**A**–**E**) Kidneys from vehicle (Veh) or NTS nephritis injured–mice (day 9) were harvested and scRNA-seq was performed. (**A**) Uniform manifold approximation and projection (UMAP) plot showing single-cell clusters (TUB: kidney tubules; PODO: podocyte; EC: endothelial cell; FIBRO: fibroblast-mesangial cells; MYELOID: monocytes, myeloid cells, and DCs; PMN: neutrophil; LYMPHO: T or B cell). Differential expression analysis followed by GO analysis was performed on cell clusters between Veh and day 9 NTS samples. Shown in graphs are upregulated GO Biological Processes for (**B**) TUB, (**C**) FIBRO, and (**D**) ENDO (EC) clusters. (**E**) Dot plot shows expression of *Irf7* in kidney cell clusters between Veh and day 9 NTS samples. (**F**–**J**) MWT and MKO mice were subjected to NTS nephritis, and individual kidney cell populations were sorted per schematic in **F** (created with BioRender.com). RT-PCR was performed and box plots show mRNA levels of *Ifnb1* in (**G**) endothelial cells, (**H**) tubule cells, (**I**) fibroblasts, and (**J**) CD11b^+^ myeloid cells (*n* = 5; *P* < 0.05 by unpaired 2-tailed *t* test). Squares represent male animals.

**Figure 4:**
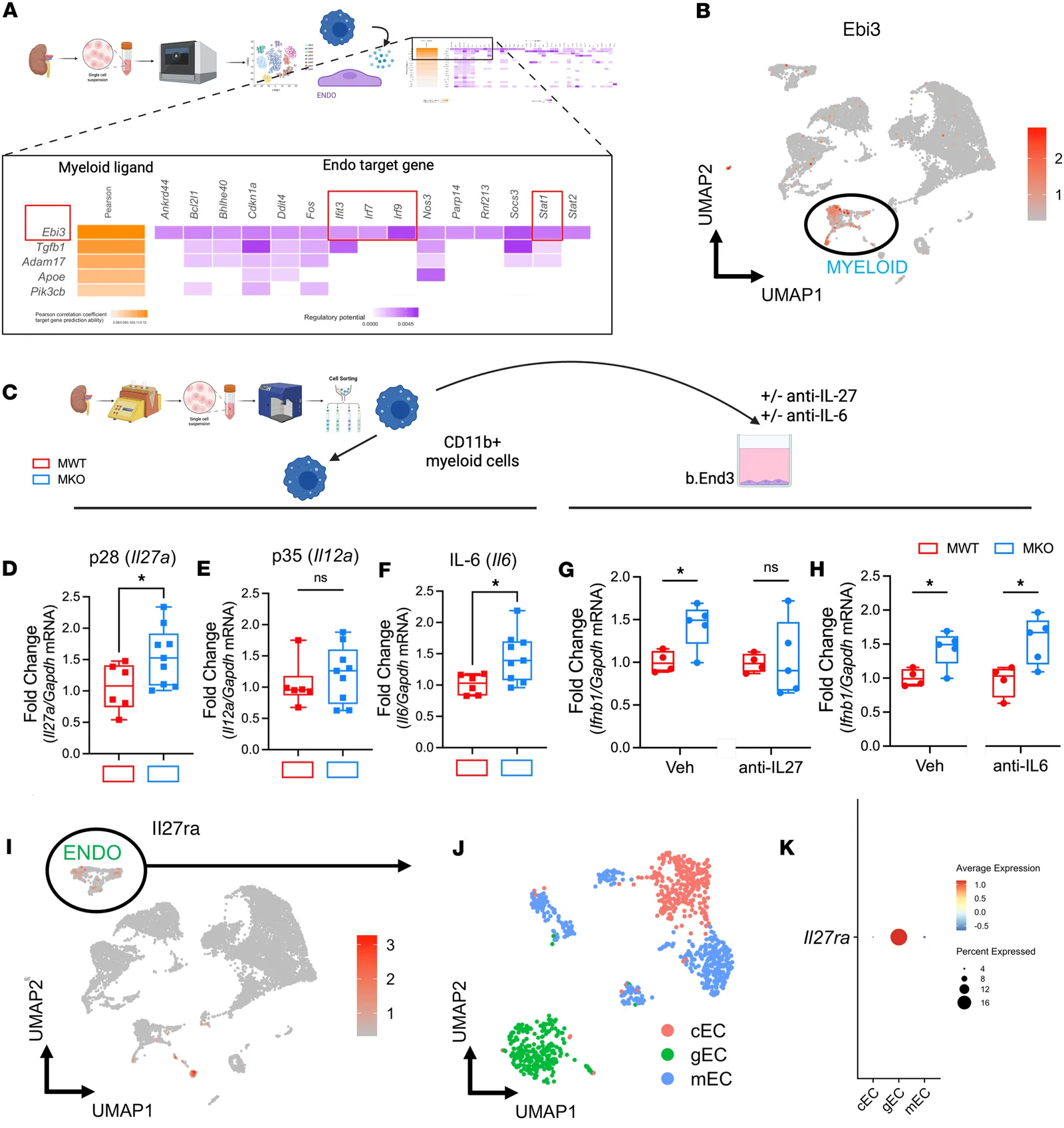
Myeloid IL-1R1 deletion increases type I IFN expression in endothelial cells after NTS nephritis: possible role of Ebi3/IL-27. (**A**) NicheNet analysis of ligand-target gene interaction with myeloid cluster as sender and endothelial cluster as receiving cells. Callout shows 5 highest prioritized ligands from myeloid cluster that induce target genes in endothelial cluster. Red boxes indicate *Ebi3* in myeloid cluster and type I IFN–associated genes in endothelial cluster (created with BioRender.com). (**B**) Feature plot showing expression of *Ebi3* mainly in myeloid cluster (circled). (**C**–**H**) MWT and MKO mice were subjected to NTS nephritis, and CD11b^+^ myeloid cells were sorted as shown in schematic (created with BioRender.com). (**D**–**F**) RT-PCR was performed, and box plots show expression of EBI3 binding partners (**D**) p28 (*Il27a*), (**E**) p35 (*Il12a*), and (**F**) IL-6 (*Il6*) (*n* = 6–9; *P < 0.05 by unpaired 2-tailed *t* test). (**G** and **H**) Myeloid cells were cocultured with brain endothelioma (b.End3) cells in presence or absence of (**G**) anti–IL-27 or (**H**) anti–IL-6. *Ifnb1* levels were measured in b.End3 cells by RT-PCR and are shown in box plots (*n* = 4–5; *P < 0.05, **P < 0.01 by unpaired 2-tailed *t* test). (**I**) Feature plot showing expression of *Il27ra* mainly in endothelial cluster (circled). (**J**) UMAP plot showing endothelial cells subclustered into cortical (cEC), glomerular (gEC), or medullary (mEC) clusters. (**K**) Dot plot showing expression of *Il27ra* in EC subclusters. Circles represent female animals and squares represent male animals.

**Figure 5:**
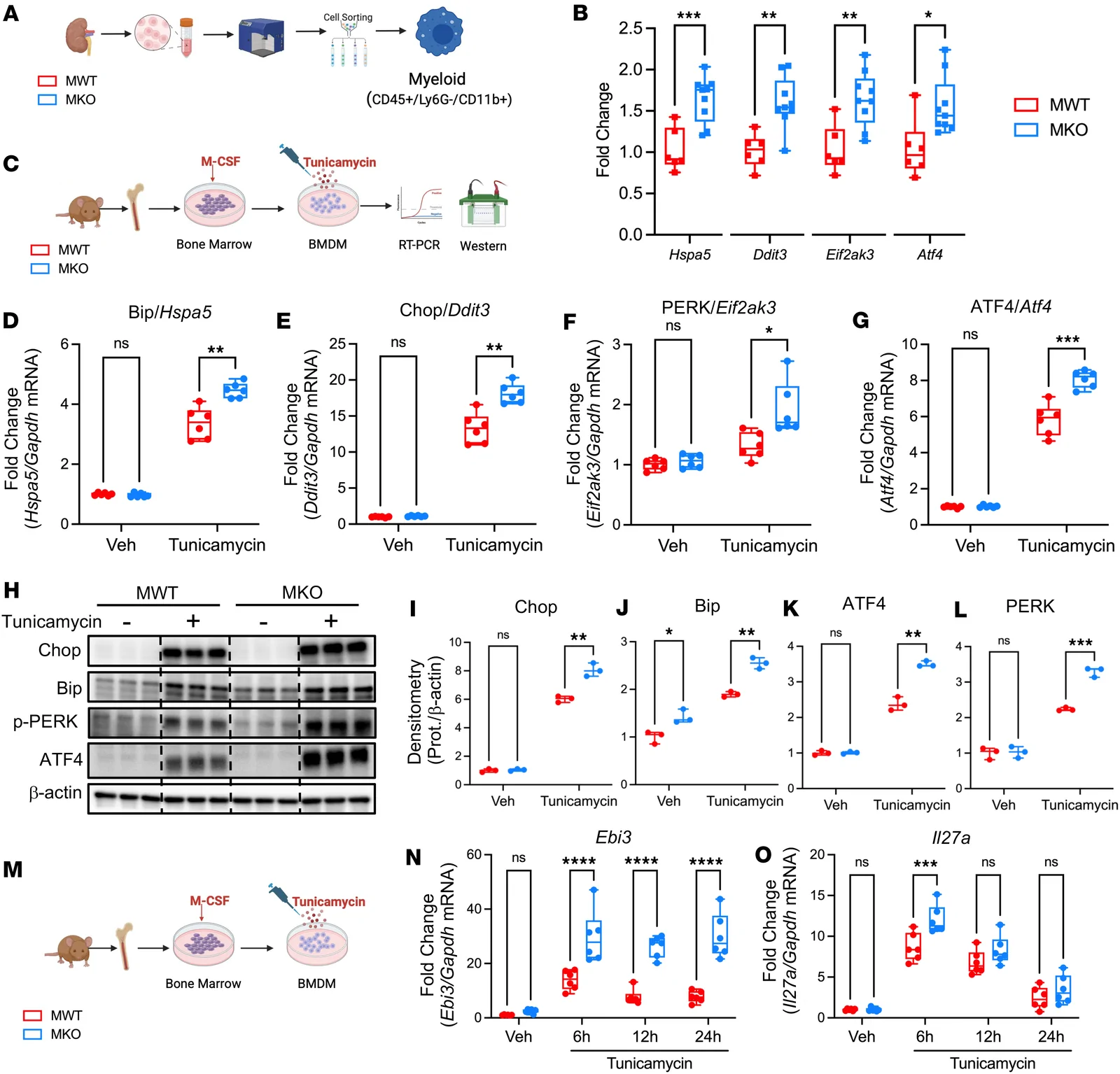
Myeloid IL-1R1 deletion increases ER stress. (**A** and **B**) (**A**) Schematic of the isolation and sorting of CD45^+^Ly6G^-^CD11b^+^ myeloid cells from the kidneys of MWT and MKO mice after NTS nephritis. (**B**) RT-PCR analysis of mRNA levels of the ER stress genes *Hspa5* (BiP), *Ddit3* (CHOP), *Eif2ak3* (PERK), and *Atf4* (ATF4) (*n* = 6–9; *P < 0.05, **P < 0.01, ***P < 0.001 by unpaired 2-tailed *t* test). (**C**–**L**) Experimental workflow of BMDMs treated with ER stress inducer tunicamycin. RT-PCR was performed, and box plots show mRNA levels of (**D**) *Hspa5*, (**E**) *Ddit3*, (**F**) *Eif2ak3*, and (**G**) *Atf4*. (**H**) Representative Western blot with box plots showing densitometry analysis for (**I**) CHOP, (**J**) BiP, (**K**) ATF4, and (**L**) phosphorylated PERK (*n* = 3; *P < 0.05, **P < 0.01 by 2-way ANOVA with Bonferroni’s post hoc test). (**M**–**O**) BMDMs from MWT and MKO mice were stimulated with tunicamycin for various time points, and box plots show mRNA levels of (**N**) *Ebi3* and (**O**) *Il27a* measured by RT-PCR (*n* = 6/group, ***P < 0.001, ****P < 0.0001 by 2-way ANOVA with Bonferroni’s posttest). Circles represent female animals and squares represent male animals. Schematics in **A**, **C**, and **M** created with BioRender.com.

**Figure 6:**
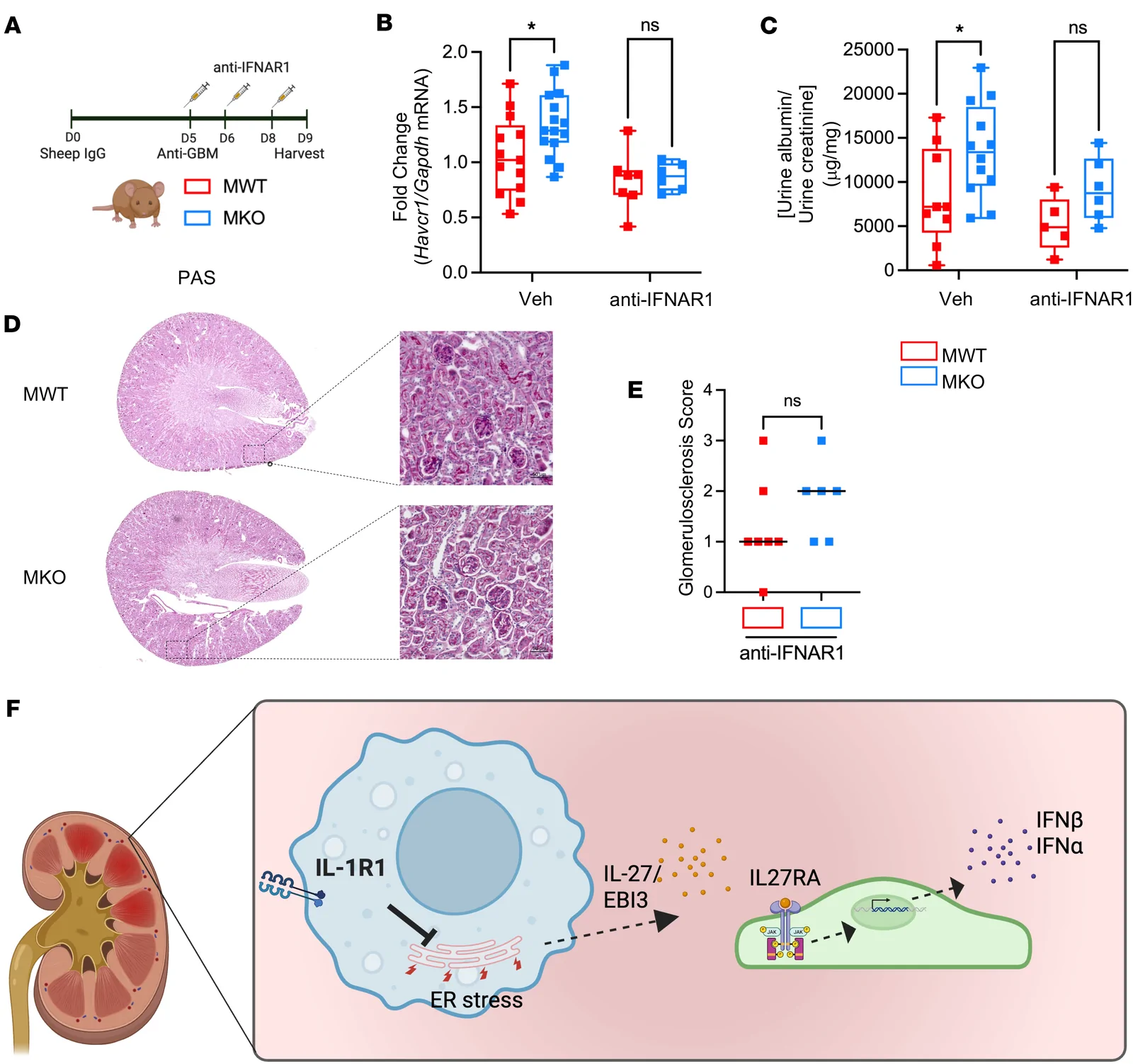
Anti-IFNAR1 therapy abrogates exaggerated albuminuria and kidney injury in MKO mice after NTS nephritis. (**A**) Schematic of experimental model of MWT and MKO subjected to NTS induction in presence or absence of anti-IFNAR1 treatment (day 5, 500 μg; day 6, 500 μg; day 8, 250 μg). Shown in box plots are (**B**) mRNA expression of KIM-1 in injured kidneys (*n* = 13–15 for vehicle groups; *n* = 6–7 for anti-IFNAR1 groups) and (**C**) urine albuminuria (urine albumin normalized to urine creatinine) (*n* = 9–12 for vehicle groups; *n* = 5–6 for anti-IFNAR1 groups; *P < 0.05, **P < 0.01 by 2-way ANOVA with Bonferroni’s post hoc test). (**D**) Representative PAS-stained kidney sections and higher magnification images are shown. (**E**) Glomerular injury was assessed by blinded observer. Each dot represents 1 mouse with line at median (*n* = 6–7 per group; statistical significance was determined by Mann-Whitney *U* test). Circles represent female animals and squares represent male animals. (**F**) Schematic of model where myeloid IL-1R1 constrains ER stress–mediated EBI3/IL27 secretion, thereby attenuating type I IFN expression in kidney endothelial cells and ameliorating autoimmune nephritis (created with BioRender.com).

## Version 1 09/03/2026

In-Press Preview

## Version 2 10/08/2026

Electronic publication

## References

[1] Jager KJ (2019). A single number for advocacy and communication-worldwide more than 850 million individuals have kidney diseases. Kidney Int.

[2] Drawz P, Rahman M (2015). Chronic kidney disease. Ann Intern Med.

[3] Meng XM (2014). Inflammatory processes in renal fibrosis. Nat Rev Nephrol.

[4] Ruiz-Ortega M (2020). Targeting the progression of chronic kidney disease. Nat Rev Nephrol.

[5] Davidson A (2016). What is damaging the kidney in lupus nephritis?. Nat Rev Rheumatol.

[6] Patrick DM (2022). Isolevuglandins disrupt PU.1-mediated C1q expression and promote autoimmunity and hypertension in systemic lupus erythematosus. JCI Insight.

[7] Haq M (1998). Role of IL-1 in renal ischemic reperfusion injury. J Am Soc Nephrol.

[8] Jones LK (2009). IL-1RI deficiency ameliorates early experimental renal interstitial fibrosis. Nephrol Dial Transplant.

[9] Zhang JD (2014). Type 1 angiotensin receptors on macrophages ameliorate IL-1 receptor-mediated kidney fibrosis. J Clin Invest.

[10] Yang B (2019). Interleukin-1 receptor activation aggravates autosomal dominant polycystic kidney disease by modulating regulated necrosis. Am J Physiol Renal Physiol.

[11] Privratsky JR (2018). Interleukin 1 receptor (IL-1R1) activation exacerbates toxin-induced acute kidney injury. Am J Physiol Renal Physiol.

[12] Ren J (2023). Divergent actions of renal tubular and endothelial type 1 IL-1 receptor signaling in toxin-induced AKI. J Am Soc Nephrol.

[13] Anders HJ (2016). Of inflammasomes and alarmins: IL-1β and IL-1α in kidney disease. J Am Soc Nephrol.

[14] Chen Y (2024). Novel anti-inflammatory effects of the IL-1 receptor in kidney myeloid cells following ischemic AKI. Front Mol Biosci.

[15] Ren J (2022). IL-1 receptor signaling in podocytes limits susceptibility to glomerular damage. Am J Physiol Renal Physiol.

[16] Meka RR (2015). IL-27-induced modulation of autoimmunity and its therapeutic potential. Autoimmun Rev.

[17] Lucas S (2003). IL-27 regulates IL-12 responsiveness of naive CD4^+^ T cells through Stat1-dependent and -independent mechanisms. Proc Natl Acad Sci U S A.

[18] Pflanz S (2002). IL-27, a heterodimeric cytokine composed of EBI3 and p28 protein, induces proliferation of naive CD4^+^ T cells. Immunity.

[19] Cao Y (2014). IL-27, a cytokine, and IFN-λ1, a type III IFN, are coordinated to regulate virus replication through type I IFN. J Immunol.

[20] Chen Q (2013). Interleukin-27 is a potent inhibitor of cis HIV-1 replication in monocyte-derived dendritic cells via a type I interferon-independent pathway. PLoS One.

[21] Greenwell-Wild T (2009). Interleukin-27 inhibition of HIV-1 involves an intermediate induction of type I interferon. Blood.

[22] Imamichi T (2008). IL-27, a novel anti-HIV cytokine, activates multiple interferon-inducible genes in macrophages. AIDS.

[23] Kwock JT (2020). IL-27 signaling activates skin cells to induce innate antiviral proteins and protects against Zika virus infection. Sci Adv.

[24] Isaacs A, Lindenmann J (1957). Virus interference. I. The interferon. Proc R Soc Lond B Biol Sci.

[25] McNab F (2015). Type I interferons in infectious disease. Nat Rev Immunol.

[26] Dunn GP (2005). A critical function for type I interferons in cancer immunoediting. Nat Immunol.

[27] Der E (2019). Tubular cell and keratinocyte single-cell transcriptomics applied to lupus nephritis reveal type I IFN and fibrosis relevant pathways. Nat Immunol.

[28] Kato Y (2018). Apoptosis-derived membrane vesicles drive the cGAS-STING pathway and enhance type I IFN production in systemic lupus erythematosus. Ann Rheum Dis.

[29] Lodi L (2022). Type I interferon-related kidney disorders. Kidney Int.

[30] Tumlin J (2025). Targeting the type I interferon pathway in glomerular kidney disease: rationale and therapeutic opportunities. Kidney Int Rep.

[31] Park Y (2025). Myeloid cells in acute kidney injury. Semin Nephrol.

[32] Lee S (2011). Distinct macrophage phenotypes contribute to kidney injury and repair. J Am Soc Nephrol.

[33] Zhang MZ (2012). CSF-1 signaling mediates recovery from acute kidney injury. J Clin Invest.

[34] Saito S (2025). Adverse functions of neutrophils are regulated by neutrophilic angiotensin-converting enzyme in immune complex-mediated crescentic glomerulonephritis. Am J Physiol Renal Physiol.

[35] Xu L (2022). Immune-mediated tubule atrophy promotes acute kidney injury to chronic kidney disease transition. Nat Commun.

[36] Lech M (2014). Macrophage phenotype controls long-term AKI outcomes--kidney regeneration versus atrophy. J Am Soc Nephrol.

[37] Nakazawa D (2018). Extracellular traps in kidney disease. Kidney Int.

[38] Stamatiades EG (2016). Immune monitoring of trans-endothelial transport by kidney-resident macrophages. Cell.

[39] Privratsky JR (2023). A macrophage-endothelial immunoregulatory axis ameliorates septic acute kidney injury. Kidney Int.

[40] Xie C (2004). Strain distribution pattern of susceptibility to immune-mediated nephritis. J Immunol.

[41] Kirita Y (2020). Cell profiling of mouse acute kidney injury reveals conserved cellular responses to injury. Proc Natl Acad Sci U S A.

[42] Park J (2018). Single-cell transcriptomics of the mouse kidney reveals potential cellular targets of kidney disease. Science.

[43] Clark JZ (2019). Representation and relative abundance of cell-type selective markers in whole-kidney RNA-seq data. Kidney Int.

[44] Honda K (2005). IRF-7 is the master regulator of type-I interferon-dependent immune responses. Nature.

[45] Browaeys R (2020). NicheNet: modeling intercellular communication by linking ligands to target genes. Nat Methods.

[46] Artis D (2004). The IL-27 receptor (WSX-1) is an inhibitor of innate and adaptive elements of type 2 immunity. J Immunol.

[47] Batten M (2006). Interleukin 27 limits autoimmune encephalomyelitis by suppressing the development of interleukin 17-producing T cells. Nat Immunol.

[48] Do J (2017). Treg-specific IL-27Rα deletion uncovers a key role for IL-27 in Treg function to control autoimmunity. Proc Natl Acad Sci U S A.

[49] Stumhofer JS (2006). Interleukin 27 negatively regulates the development of interleukin 17-producing T helper cells during chronic inflammation of the central nervous system. Nat Immunol.

[50] Takeda A (2003). Cutting edge: role of IL-27/WSX-1 signaling for induction of T-bet through activation of STAT1 during initial Th1 commitment. J Immunol.

[51] Clausen BE (1999). Conditional gene targeting in macrophages and granulocytes using LysMcre mice. Transgenic Res.

[52] Zhang T (2022). Epstein-Barr virus-induced gene 3 commits human mesenchymal stem cells to differentiate into chondrocytes via endoplasmic reticulum stress sensor. PLoS One.

[53] Deng B (2021). Plasmacytoid dendritic cells promote acute kidney injury by producing interferon-α. Cell Mol Immunol.

[54] De Cecco M (2019). L1 drives IFN in senescent cells and promotes age-associated inflammation. Nature.

[55] Migliorini A (2013). The antiviral cytokines IFN-α and IFN-β modulate parietal epithelial cells and promote podocyte loss: implications for IFN toxicity, viral glomerulonephritis, and glomerular regeneration. Am J Pathol.

[56] Fairhurst AM (2009). Type I interferons produced by resident renal cells may promote end-organ disease in autoantibody-mediated glomerulonephritis. J Immunol.

[57] Nacionales DC (2007). Deficiency of the type I interferon receptor protects mice from experimental lupus. Arthritis Rheum.

[58] Lee J (2021). Baricitinib attenuates autoimmune phenotype and podocyte injury in a murine model of systemic lupus erythematosus. Front Immunol.

[59] Davis SE (2019). Nucleosomal dsDNA stimulates APOL1 expression in human cultured podocytes by activating the cGAS/IFI16-STING signaling pathway. Sci Rep.

[60] Nichols B (2015). Innate immunity pathways regulate the nephropathy gene Apolipoprotein L1. Kidney Int.

[61] Pays E (2024). The mechanism of kidney disease due to APOL1 risk variants: involvement of two distinct processes. J Am Soc Nephrol.

[62] Wu J (2021). The key role of NLRP3 and STING in APOL1-associated podocytopathy. J Clin Invest.

[63] Congy-Jolivet N (2022). Monocytes are the main source of STING-mediated IFN-α production. EBioMedicine.

[64] Akira S (2006). Pathogen recognition and innate immunity. Cell.

[65] Muller SI (2019). A folding switch regulates interleukin 27 biogenesis and secretion of its α-subunit as a cytokine. Proc Natl Acad Sci U S A.

[66] Shimizu M (2006). Antiangiogenic and antitumor activities of IL-27. J Immunol.

[67] Feng XM (2007). Interleukin-27 upregulates major histocompatibility complex class II expression in primary human endothelial cells through induction of major histocompatibility complex class II transactivator. Hum Immunol.

[68] Qiu HN (2016). Interleukin-27 enhances TNF-α-mediated activation of human coronary artery endothelial cells. Mol Cell Biochem.

[69] Cybulsky AV (2017). Endoplasmic reticulum stress, the unfolded protein response and autophagy in kidney diseases. Nat Rev Nephrol.

[70] Chiang CK (2011). Endoplasmic reticulum stress implicated in the development of renal fibrosis. Mol Med.

[71] Cybulsky AV (2011). Endoplasmic reticulum stress in glomerular epithelial cell injury. Am J Physiol Renal Physiol.

[72] Madhusudhan T (2015). Defective podocyte insulin signalling through p85-XBP1 promotes ATF6-dependent maladaptive ER-stress response in diabetic nephropathy. Nat Commun.

[73] Dong G (2015). mTOR contributes to ER stress and associated apoptosis in renal tubular cells. Am J Physiol Renal Physiol.

[74] Shu S (2018). Endoplasmic reticulum stress is activated in post-ischemic kidneys to promote chronic kidney disease. EBioMedicine.

[75] Robson MJ (2016). Generation and characterization of mice expressing a conditional allele of the interleukin-1 receptor type 1. PLoS One.

[76] Skrypnyk NI (2013). Ischemia-reperfusion model of acute kidney injury and post injury fibrosis in mice. J Vis Exp.

[77] Macal M (2018). Self-renewal and Toll-like receptor signaling sustain exhausted plasmacytoid dendritic cells during chronic viral infection. Immunity.

[78] Xin G (2018). Single-cell RNA sequencing unveils an IL-10-producing helper subset that sustains humoral immunity during persistent infection. Nat Commun.

[79] Weischenfeldt J, Porse B (2008). Bone marrow-derived macrophages (BMM): isolation and applications. CSH Protoc.

[80] Davis BK (2013). Isolation, culture, and functional evaluation of bone marrow-derived macrophages. Methods Mol Biol.

[81] Ide S (2021). Ferroptotic stress promotes the accumulation of pro-inflammatory proximal tubular cells in maladaptive renal repair. Elife.

[82] Gerhardt LMS (2023). Lineage tracing and single-nucleus multiomics reveal novel features of adaptive and maladaptive repair after acute kidney injury. J Am Soc Nephrol.

[83] Dumas SJ (2020). Single-cell RNA sequencing reveals renal endothelium heterogeneity and metabolic adaptation to water deprivation. J Am Soc Nephrol.

