# Supplementary material for: The myeloid IL-1 receptor limits IL-27–mediated endothelial type I IFN during nephrotoxic serum nephritis
Source: JCI Insight. 2026 Sep 1;11(19):e200728. doi: 10.1172/jci.insight.200728 (PMC13726424; doi:10.1172/jci.insight.200728)
Supplement: Supplementary file 1 [file jciinsight-11-200728-s182.pdf]

Supplementary Materials for

The myeloid IL-1 receptor limits IL-27-mediated endothelial type I IFN during nephrotoxic serum nephritis

Yanting Chen *et al.*

*Corresponding author. Email: jamie.privratsky@duke.edu

This PDF file includes:

Supplemental Figures 1-14
Supplemental Tables 1-2

Supplemental Figure 1

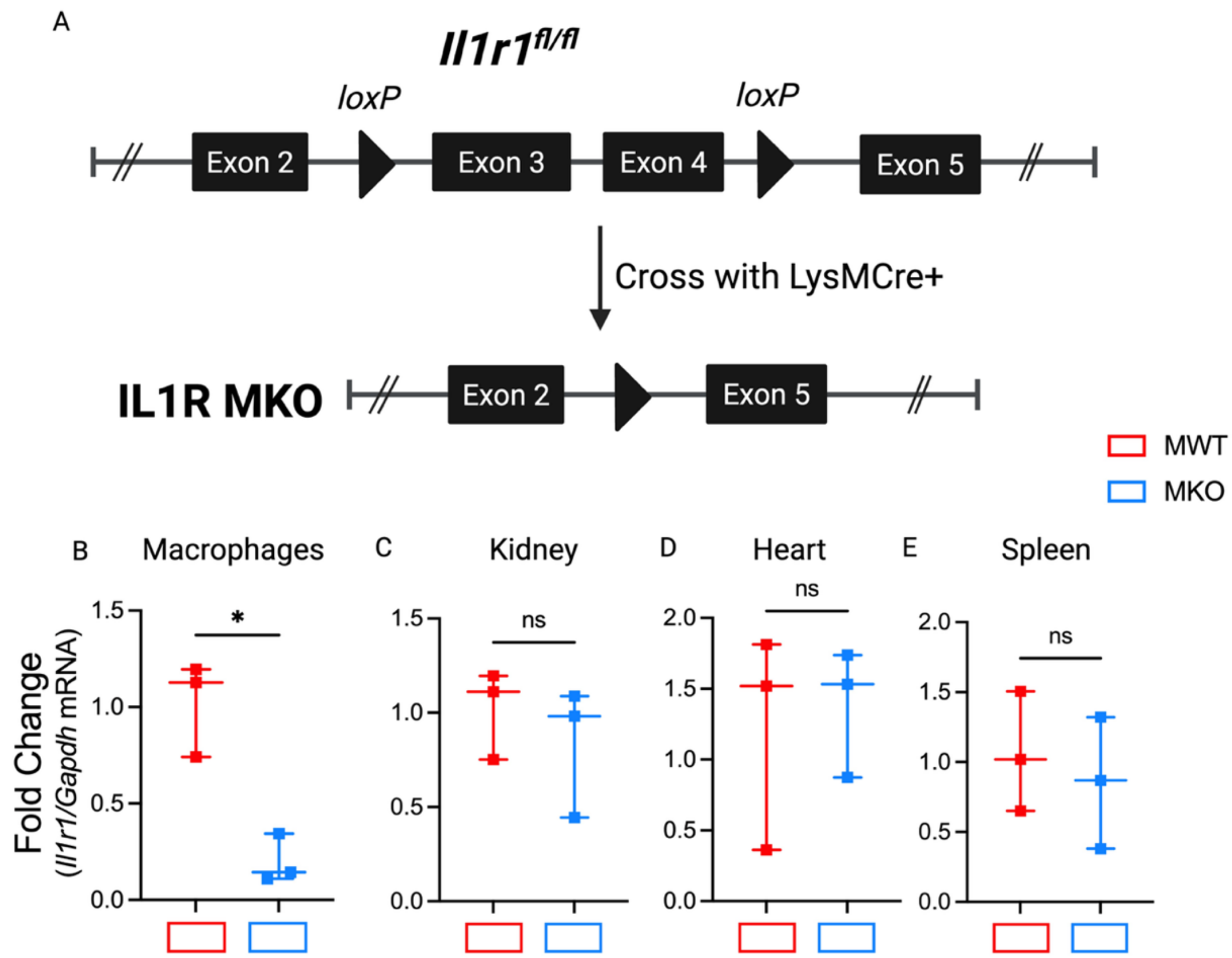

Supplemental Figure 1. Deletion of IL-1R1 in myeloid cells. (A) Schematic for tissue-specific deletion of IL1R1 in LysM+ myeloid cells (IL-1R1 MKO – *LysM-Cre(+)/IL-1R1fl/fl*). **(B-E)** Boxplots show mRNA expression of *Il1r1* across the various tissues including **(B)** peritoneal macrophages, **(C)** kidney, **(D)** heart, and **(E)** spleen ($p<0.05$ by unpaired T test).

Supplemental Figure 2

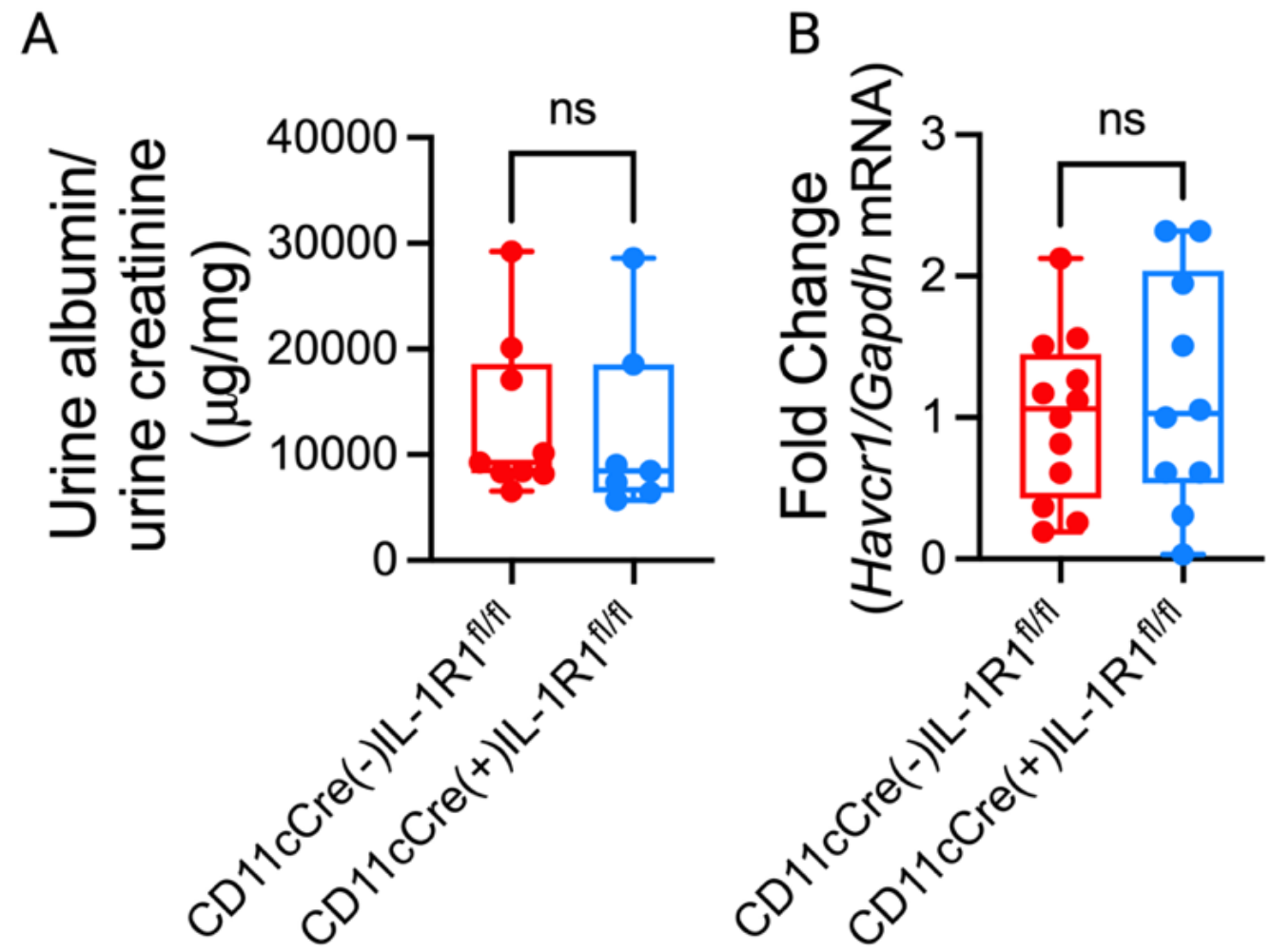

Supplemental Figure 2. CD11c-Cre IL-1R1 does not affect kidney injury in NTS nephritis. (A-B) Female *CD11c-Cre(+)/Il1r1$^{fl/fl}$* and littermate control were subjected to NTS nephritis. Boxplots show **(A)** urinary albumin excretion (normalized to urine creatinine) and **(B)** kidney *Havcr1* mRNA expression on day 9. (n = 7-9 in A, n=10-12 in B, note there are fewer animals in A as urine was not able to be collected from every animal).

Supplemental Figure 3

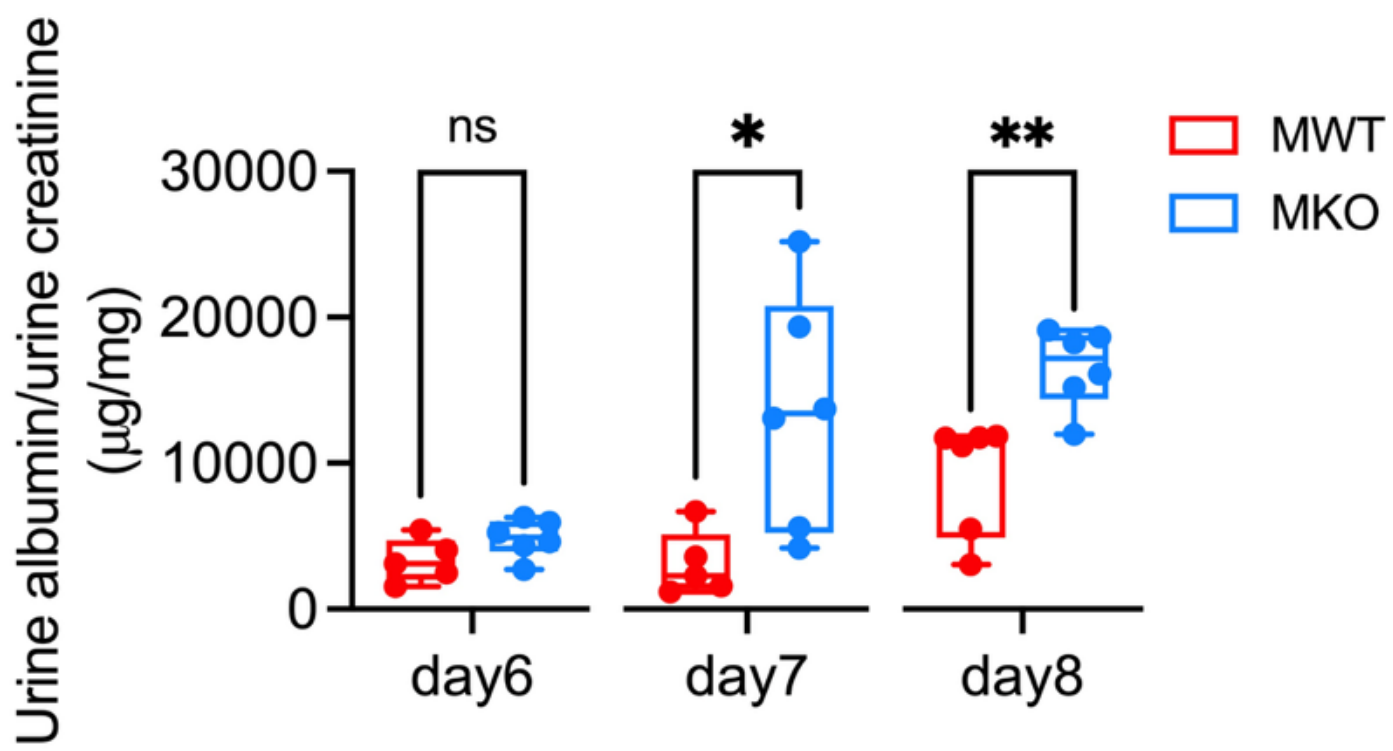

Supplemental Figure 3. Kidney injury progression following NTS nephritis. Female IL1R MWT and MKO mice were subjected to NTS induction. Boxplots show time-course analysis of urinary albumin excretion from day 6 to day 8 following NTS induction, showing progressive glomerular injury. Each dot represents one mouse (n= 5-6/group, *p < 0.05, **p < 0.01 by unpaired T tests).

Supplemental Figure 4

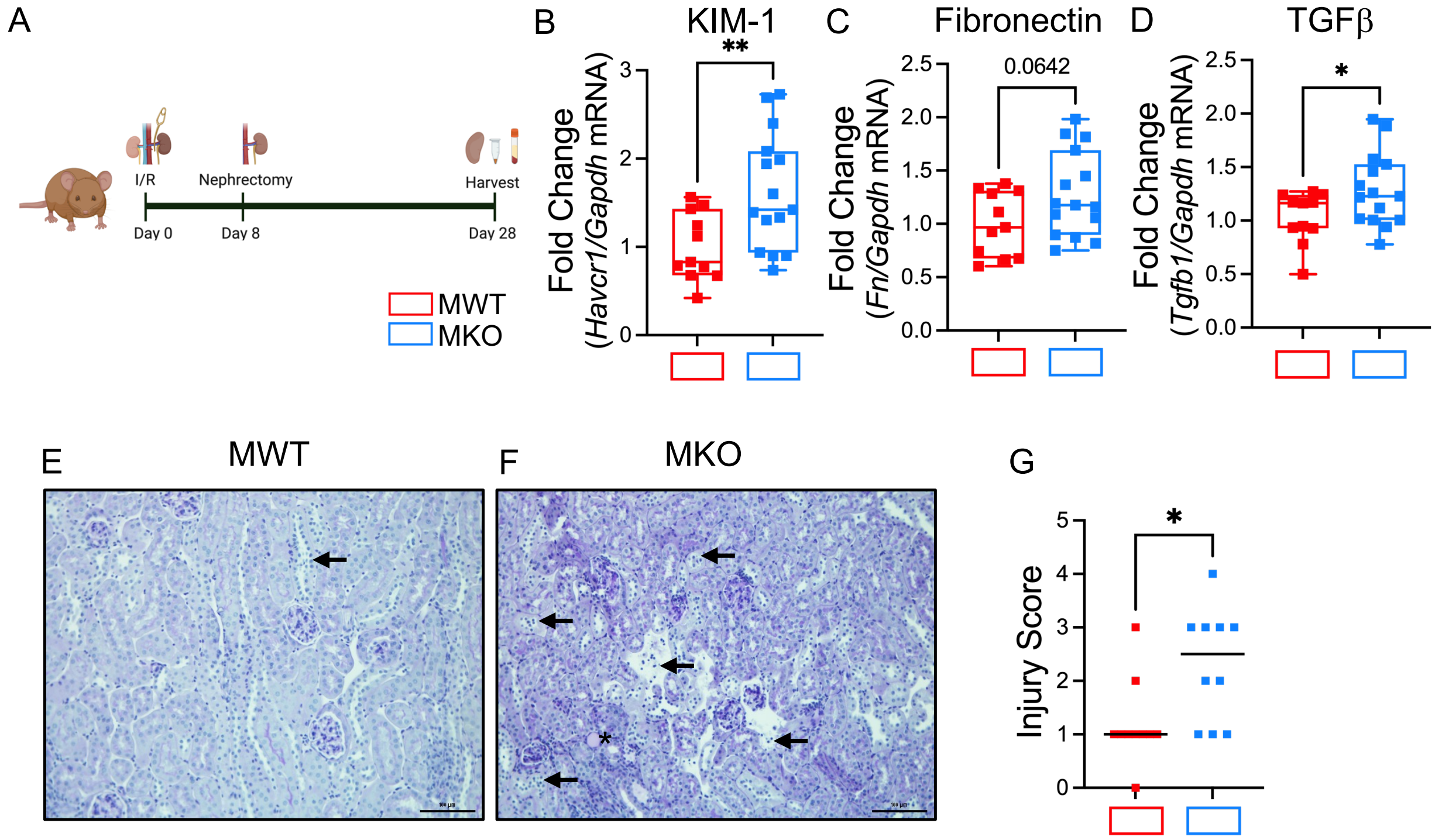

Supplemental Figure 4. **Genetic depletion of IL-1R1 on myeloid cells leads to worsened kidney tubule injury after ischemic kidney injury in mice. (A)** Schematic of experimental model of chronic ischemia/reperfusion (I/R) in male mice. Kidneys were collected on day 28 for measurement and analysis (Created with Biorender.com). **(B-D)** mRNA levels of **(B)** kidney injury molecule (KIM)-1 (*Havcr1*) and fibrosis related genes **(C)** *Fn* (fibronectin) and **(D)** *Tgfb1*. Graphs display boxplot with dots indicating individual samples (n=11-15 for I/R groups; *p<0.05, **p<0.01 by unpaired T test). **(E-F)** Representative 20x periodic-acid Schiff-stained kidney sections from **(E)** MWT and **(F)** MKO mice after chronic I/R (asterisk – casts; arrowheads – cell lysis, cells in lumen; scale bar = 100 um) with **(G)** dot plot (line at median) showing quantitation of injury scoring of kidney sections (n=10 for I/R groups; *p<0.05 by Mann-Whitney test).

Supplemental Figure 5

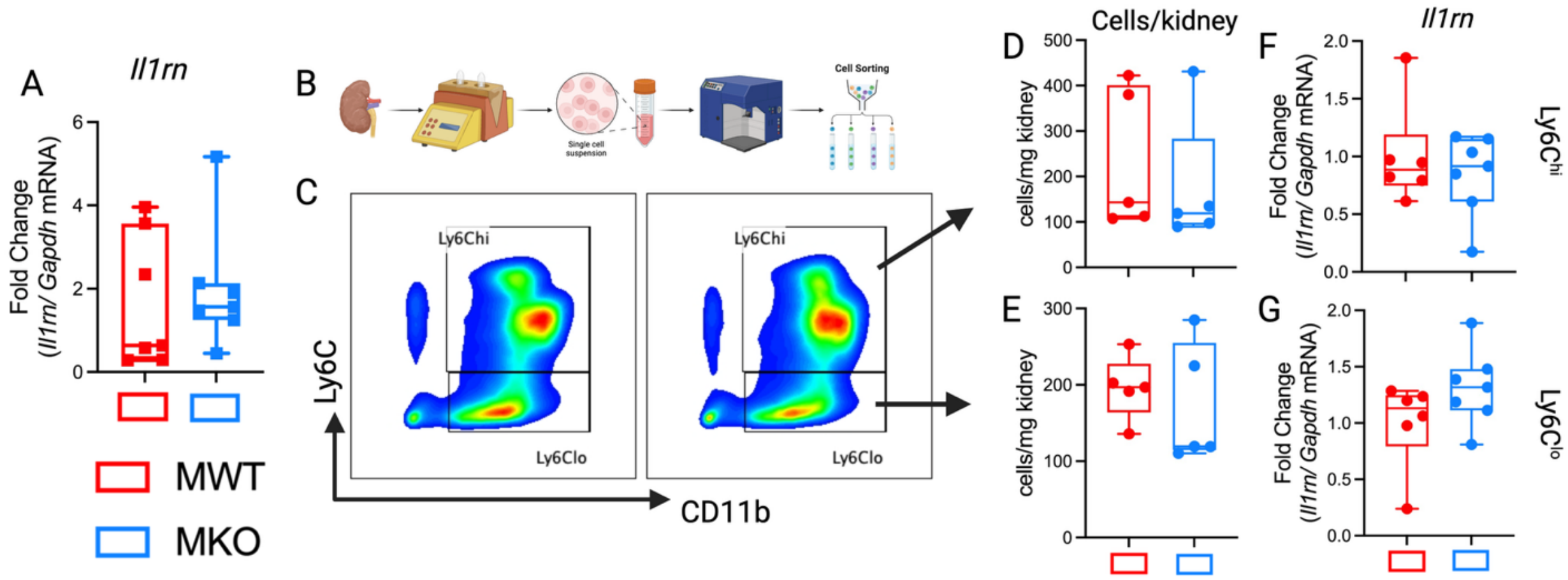

Supplemental Figure 5. *Il1rn* expression in kidney myeloid cells following NTS induction. (A) Boxplots show whole kidney mRNA levels of *Il1rn* in IL1R MWT and MKO mice at day 9 after NTS nephritis. **(B-G)** Flow cytometry and cell sorting were performed. **(C)** Representative pseudocolor dot plots of non-neutrophil myeloid cells demonstrating gates for Ly6C^{hi} and Ly6C^{lo} populations with enumeration in (**D,E**) and **(F,G)** mRNA levels of *Il1rn* in respective populations as shown in boxplots (there were no significant values as determined by unpaired T test). Created with Biorender.com. Circles represent female animals and squares represent male animals.

Supplemental Figure 6

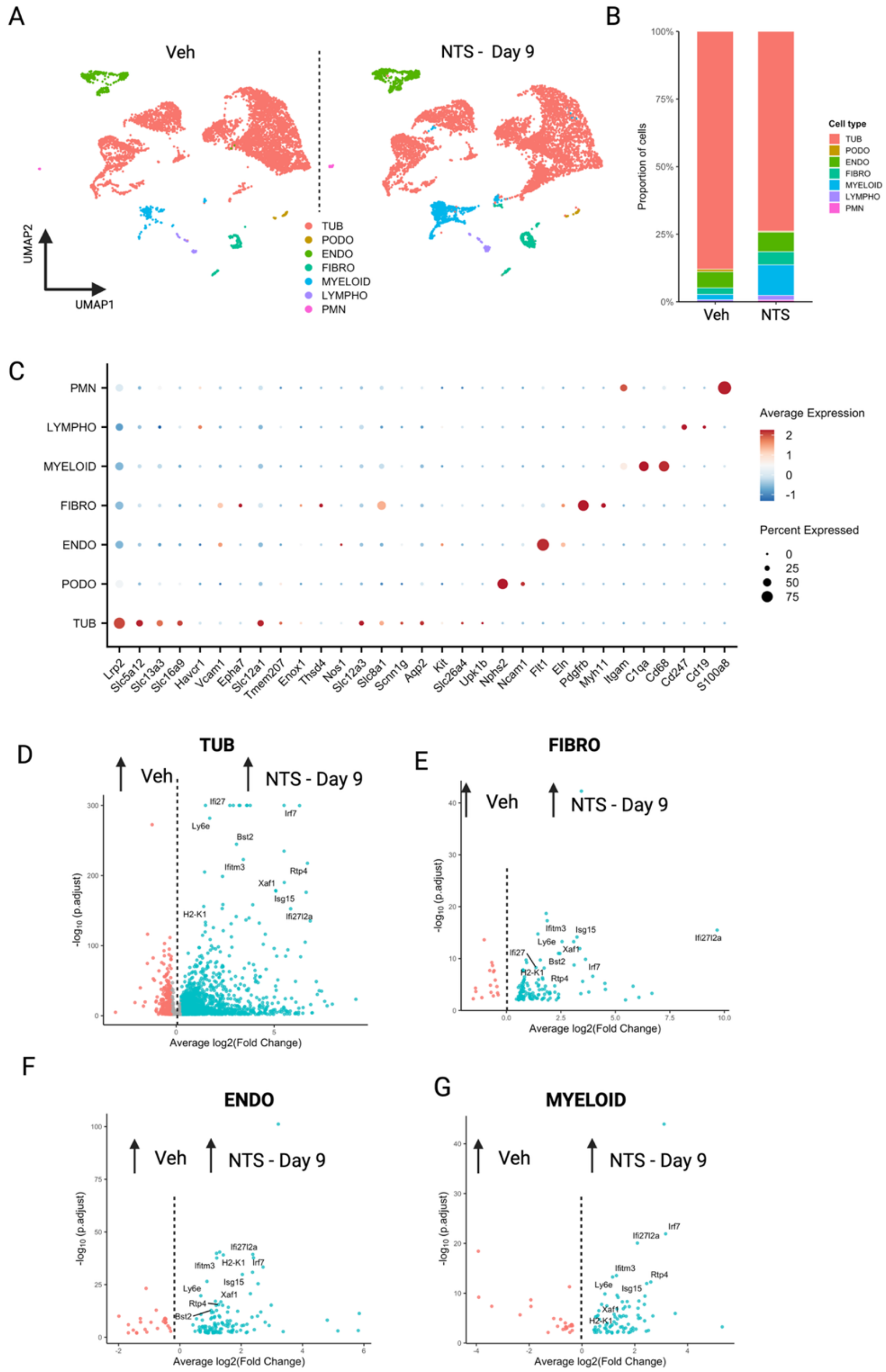

Supplemental Figure 6. Single cell RNA sequencing of kidneys of wildtype mice after NTS nephritis. Kidneys from Vehicle (Veh) or NTS nephritis injured mice (Day 9) were harvested and single cell RNA sequencing was performed. **(A)** UMAP plot showing single cell clusters with **(B)** proportions of cell populations. **(C)** Graph shows Dotplot of gene markers used to annotate cell clusters (TUB: kidney tubules; PODO: podocyte; EC: endothelial cell; FIBRO: fibroblast-mesangial cells; MYELOID: monocytes, myeloid, and dendritic cells; PMN: neutrophil; LYMPHO: T or B cell). **(D-G)** Volcano plots of differentially expressed genes between Veh vs Day 9 NTS nephritis for **(D)** TUB, **(E)** FIBRO, **(F)** ENDO, **(G)** MYELOID clusters with type I IFN-associated genes highlighted.

Supplemental Figure 7

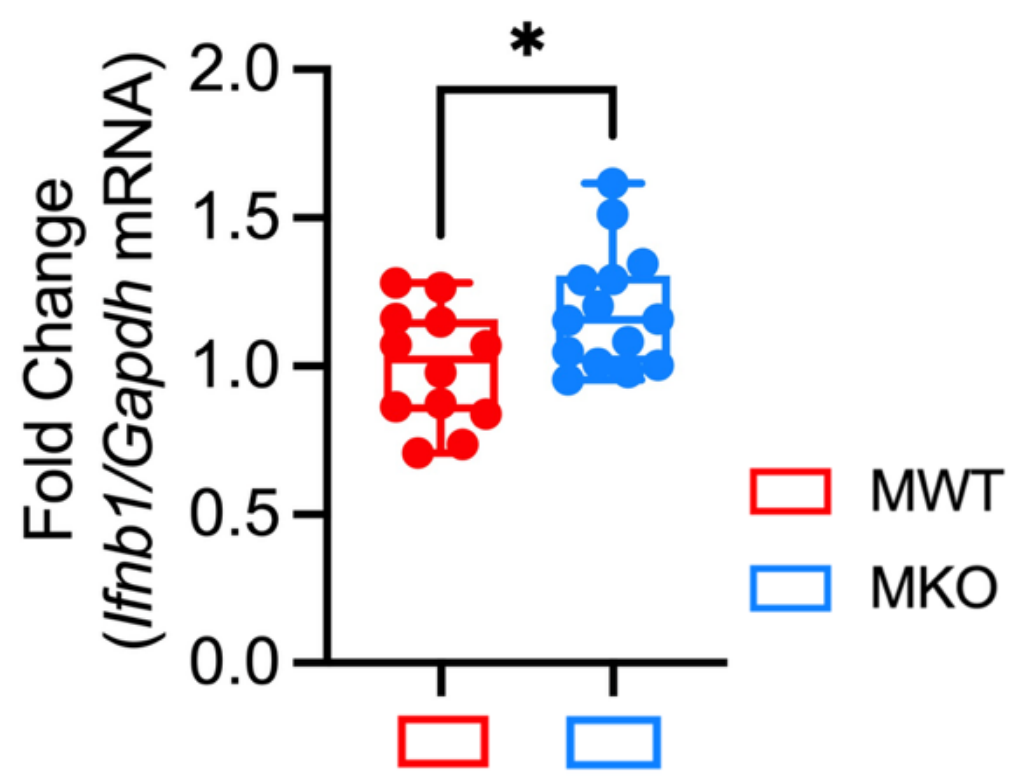

Supplemental Figure 7. Increased *Ifnb1* expression in MKO kidneys following NTS nephritis. Female MWT and MKO mice were subjected to NTS induction. Kidneys were harvested on day 9. Kidney mRNA levels of *Ifnb1* were measured in whole kidney lysates across multiple experiments. Graphs display boxplot with dots indicating individual samples (n=12-14/groups; *p<0.05 by unpaired T test).

Supplemental Figure 8

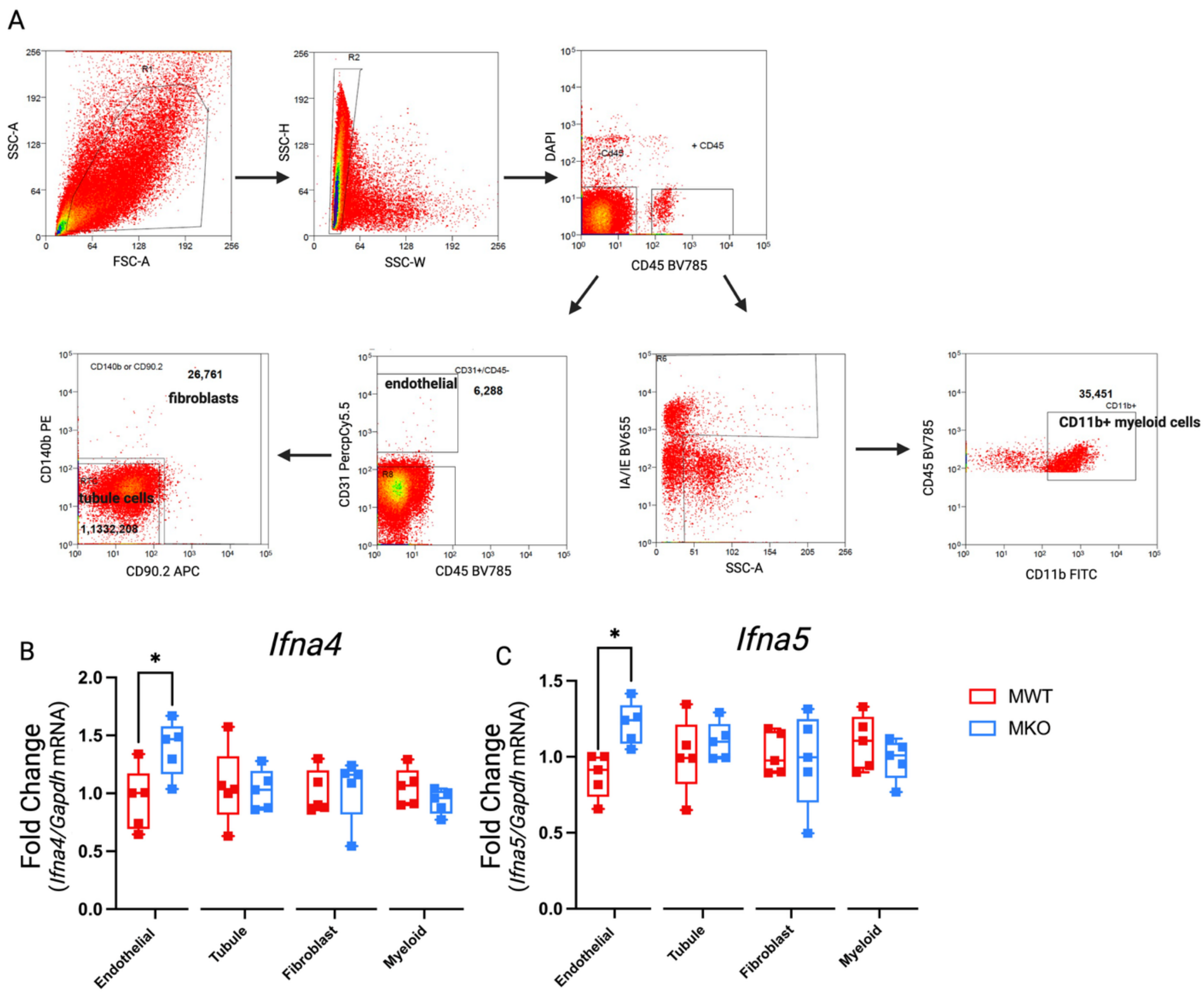

Supplemental Figure 8. Gating strategy for cell sorting and Ifna4 and Ifna5 expression in kidney cell types following NTS induction. (A) Gating strategy for sorting kidney endothelial cells, tubule cells, fibroblasts and CD11b+ myeloid cells. IL-1R1 MWT and MKO mice were subjected to NTS, and kidneys were harvested for fluorescent cell sorting. Single cells were identified based on FSC-A vs SSC-A, followed by doublet exclusion (SSC-H vs SSC-W) and live/dead cells using DAPI. Immune cells (CD45+) and non-immune cells (CD45-) were gated separately. Within the non-immune cells, fibroblasts (CD140b+/CD90.2+), tubule cells (CD140b-CD90.2-), and endothelial cells (CD31+CD45-) were identified. CD45+ cells were further gated based on MHC-II (IA/IE) and SSC-A to exclude neutrophils and CD11b+ myeloid cells were subsequently identified. **(B-C)** IL-1R1 MWT and MKO mice were subjected to NTS, and various kidney cell populations were sorted, and RT-PCR was performed for mRNA levels of (**B**) *Ifna4* and **(C)** *Ifna5* in endothelial cells, tubule cells, fibroblasts and CD11b+ myeloid cells. Graphs display boxplots with dots indicating individual samples. (n = 5; *p<0.05 by unpaired T test). Squares represent male animals.

Supplemental Figure 9

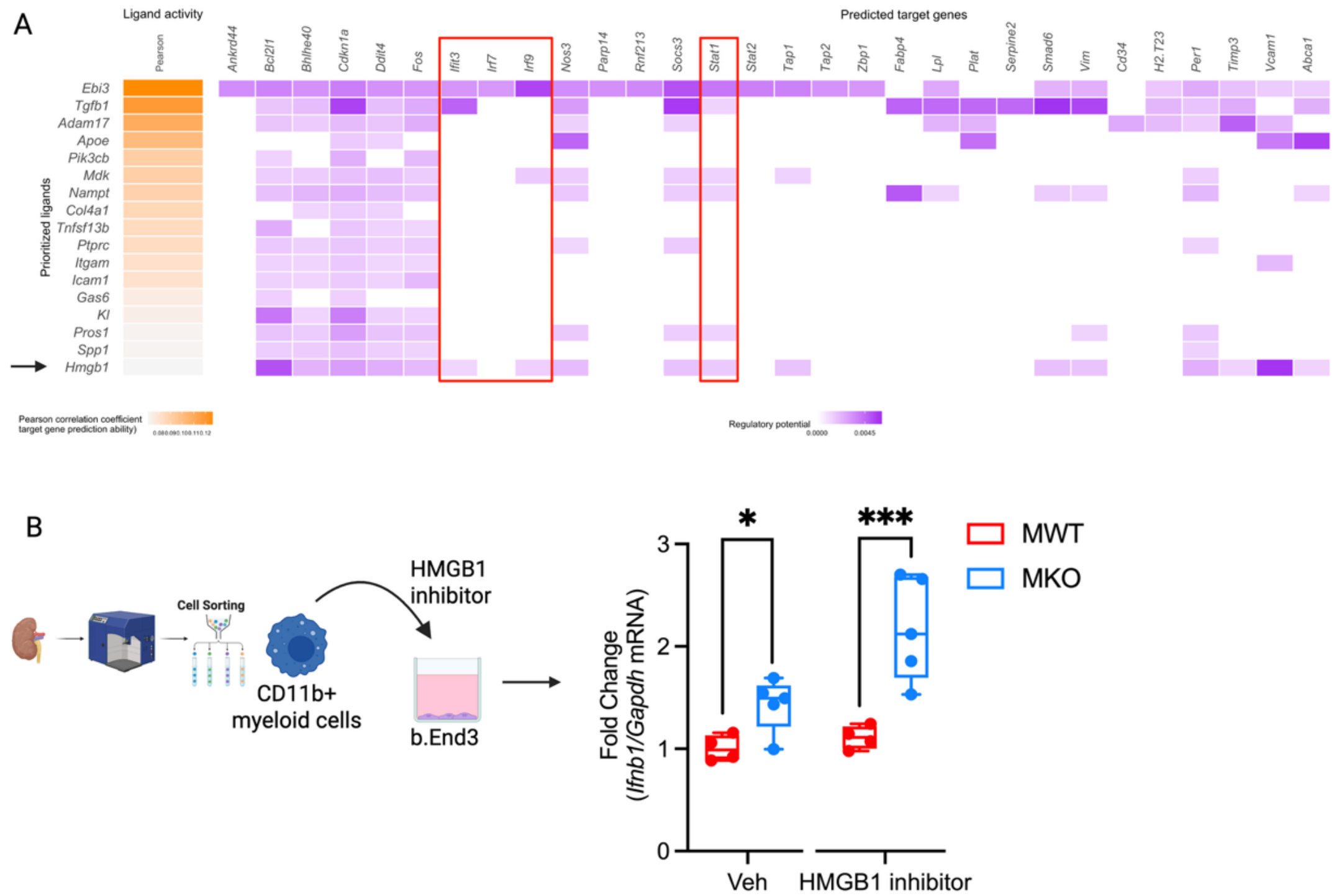

Supplemental Figure 9. HMGB1 signaling is unlikely to be the primary mediator of the elevated endothelial type I interferon response observed in MKO mice. (A) NicheNet analysis of ligand-target gene interaction with myeloid cluster as sender and endothelial cluster as receiving cells. Callout shows the prioritized ligands (left panel) from myeloid cluster that induce target genes in endothelial cluster (right panel). Arrow indicates *Hmgb1* in myeloid cluster and red box indicates type I IFN-associated target genes in endothelial cluster. **(B)** Myeloid cells were co-cultured with b.End3 cells in presence or absence of HMGB1 inhibitor (Glycyrrhizin, 250µM) for 12 hours. *Ifnb* levels were measured in b.End3 cells by RT-PCR and are displayed in boxplots. (n = 4-5; *p<0.05, ***p<0.001 by unpaired T test). Circles represent female animals. Created with Biorender.com.

Supplemental Figure 10

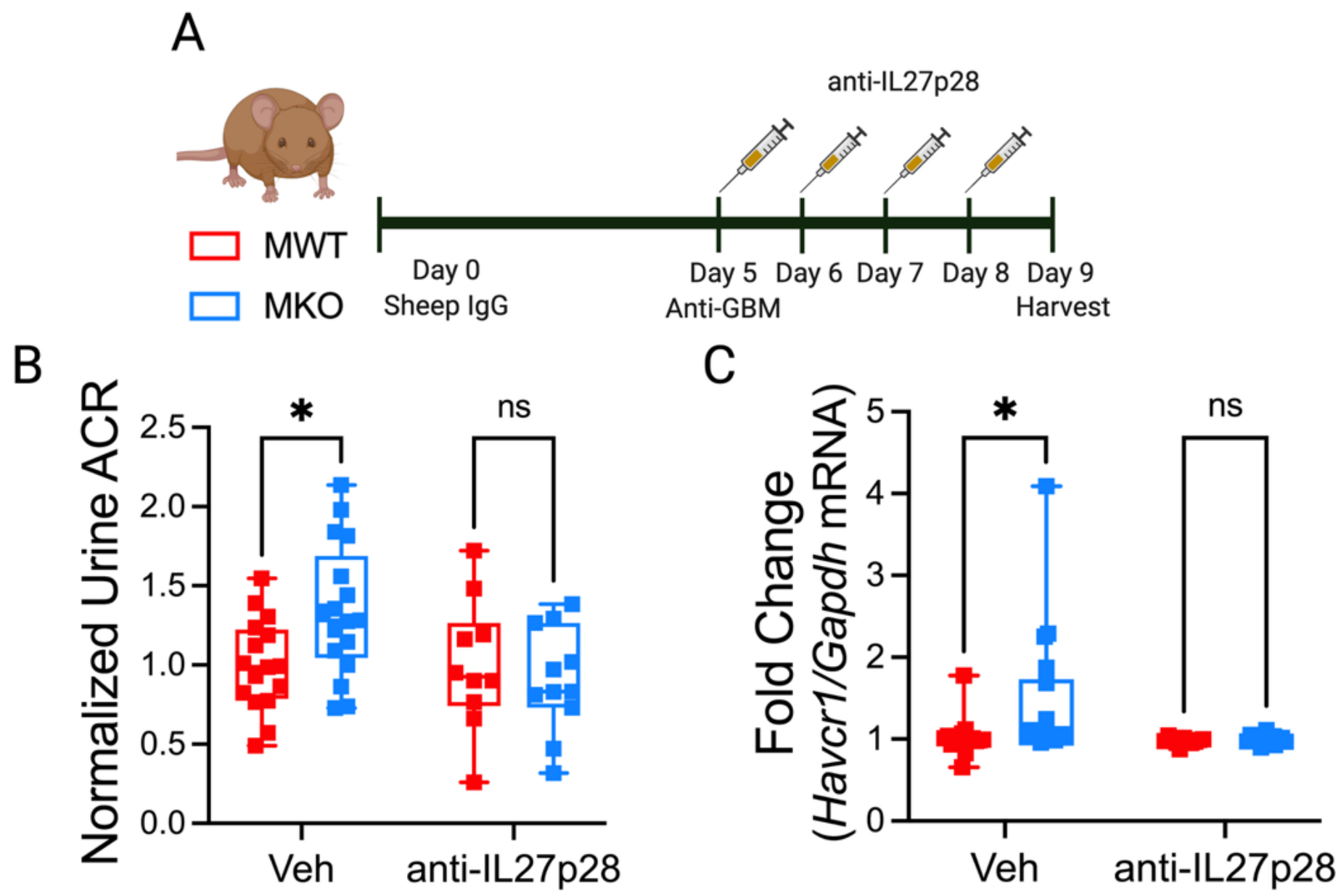

Supplemental Figure 10. IL-27 blockade attenuates albuminuria in MKO mice following NTS nephritis. (A) Experimental design. MWT and MKO mice were subjected to NTS induction and randomly assigned to receive vehicle or anti-IL-27p28 antibody treatment. Anti-IL-27p28 antibody was administered intraperitoneally on day 5, day 6, and day 7 at 500 µg/mouse, and on day 8 at 250 µg/mouse. Mice were harvested on day 9. **(B)** Urinary albumin excretion was assessed by albumin-to-creatinine ratio (ACR). Data are presented as normalized values within each experiment due to different magnitude of NTS nephritis induction between anti-GBM lots in separate experiments. Each square represents one mouse. (n=16-17 for Veh group; n= 10-11 for anti-IL27p28 group; *p<0.05 by determined by two-way ANOVA with Bonferroni post-hoc test). **(C)** Kidney mRNA levels of kidney injury marker KIM-1/*Havcr1* were measured and are displayed in boxplots. Each square represents one mouse. (n=17-18 for Veh group; n= 11-13 for anti-IL27p28 group; *p<0.05 by determined by two-way ANOVA with Bonferroni post-hoc test). Created with Biorender.com. Squares represent male animals.

Supplemental Figure 11

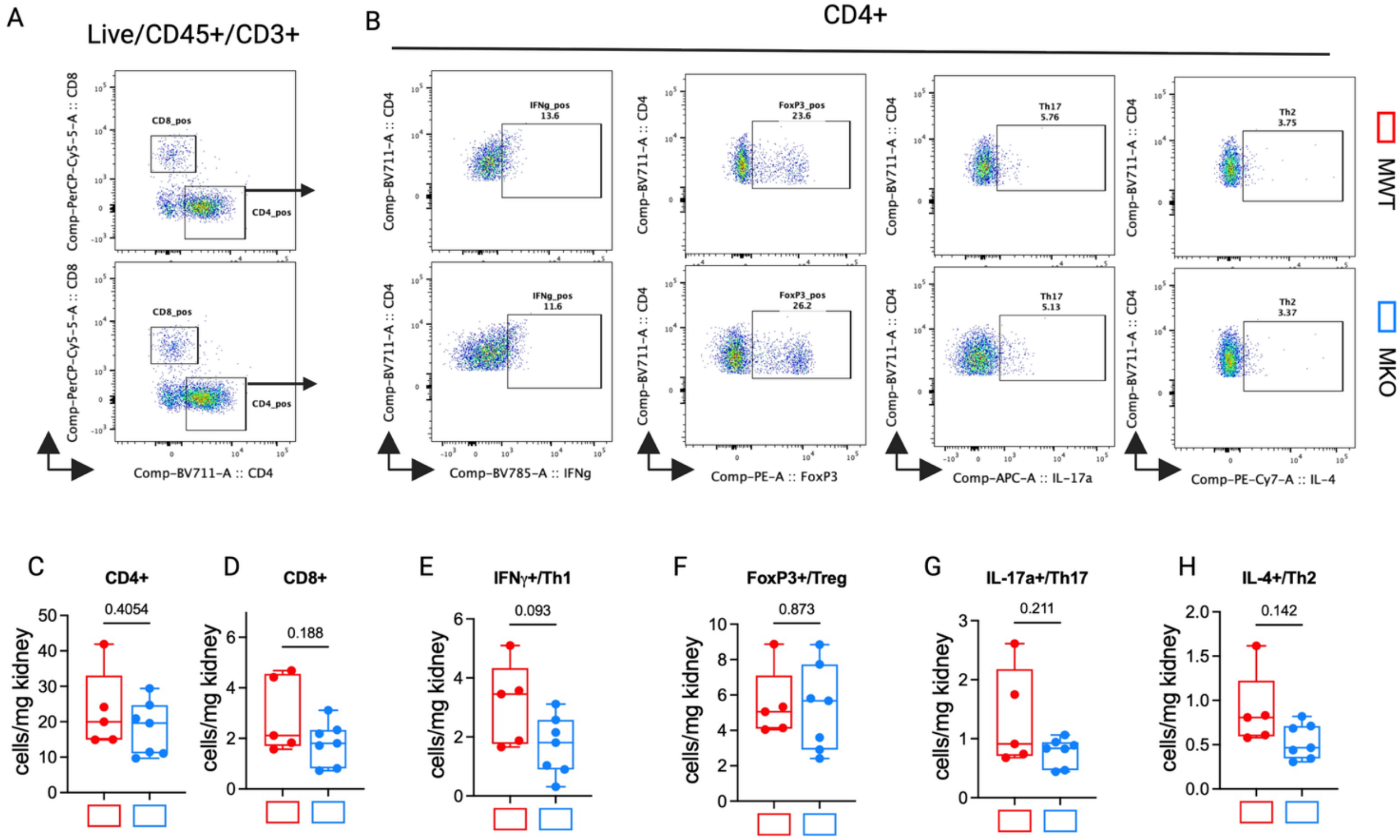

Supplemental Figure 11. IL1R MKO mice do not display changes in lymphocyte accumulation following NTS nephritis. (A-B) Kidneys from Vehicle (Veh) or NTS nephritis injured mice (Day 9) were harvested and flow cytometry was performed. We stained for cell surface markers and intracellular cytokines/transcription factors with gating strategy shown for (A) CD4 and CD8 T cells and **(B)** cytokine/transcription factor staining for CD4+ T cells. **(C-H)** Enumeration of cell populations shown in boxplots for **(C)** CD4+, **(D)** CD8+, **(E)** IFNγ+/CD4+ (Th1), **(F)** FoxP3+/CD4+ (Treg), **(G)** IL-17a+/CD4+ (Th17), and **(H)** IL-4+/CD4+ (Th2). There were no statistically significant differences between genotypes for any cell population as determined by unpaired t-tests (n = 5-7). Circles represent female animals. Created with Biorender.com.

Supplemental Figure 12

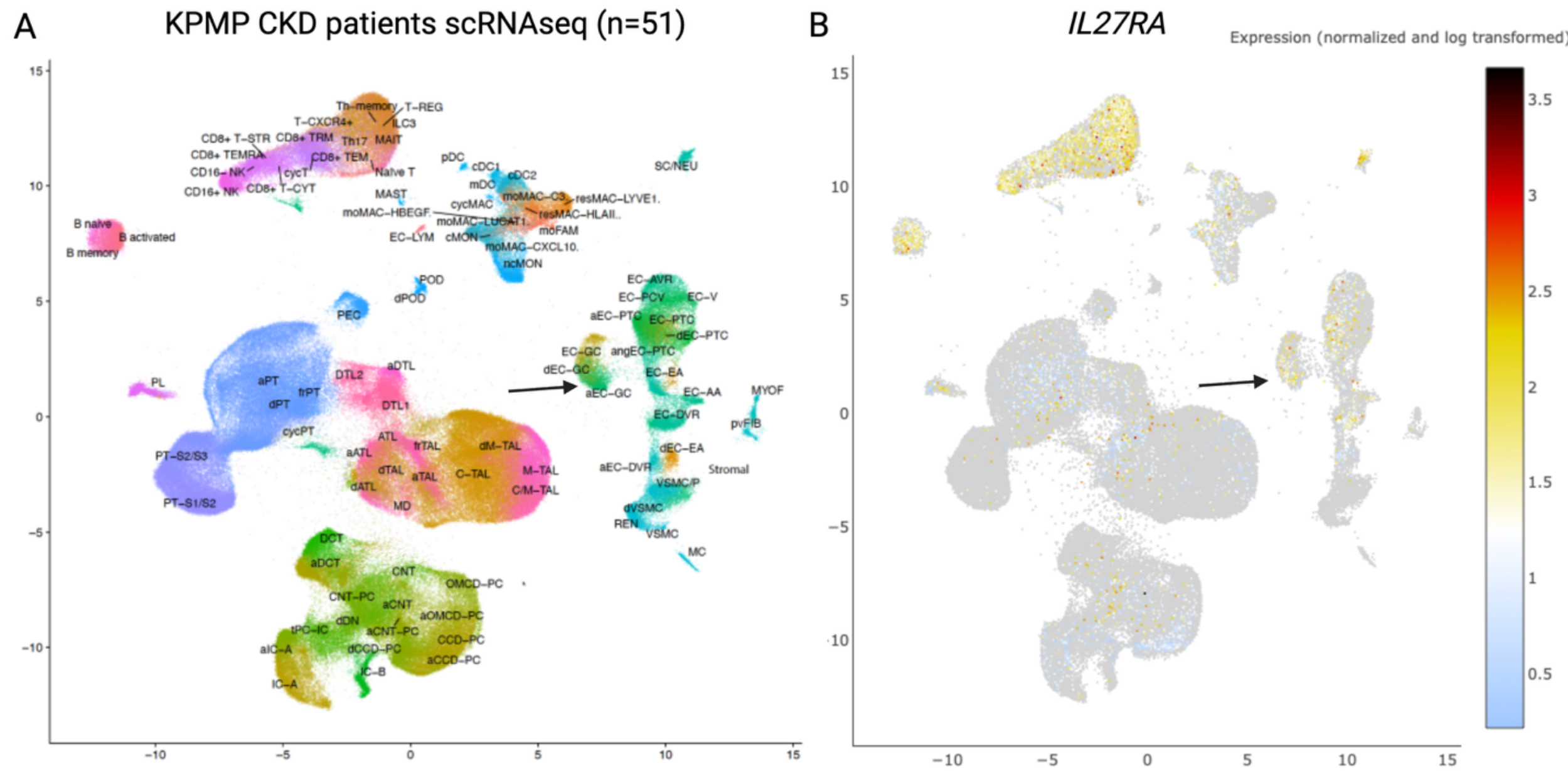

C

Abbreviation	clusterName	foldChange	pValAdj
CD8+ TEMRA	Cytotoxic T Cell, Temra (Terminally Differentiated) CD8+	2.23	1.43E-30
CD16+ NK	Natural Killer Cell, CD16+	2.62	1.83E-24
Th-memory	T Helper memory Cell	2.21	1.18E-15
MON/cMON	Classical Monocyte	1.45	2.91E-14
cDC2	Classical Type 2 Dendritic Cell	1.17	1.62E-12
B memory	B Memory Cell	2.09	5.98E-12
Th17	T Helper Cell, Type 17	2.7	2.65E-10
T-REG	Regulatory T Cell	2.25	1.04E-09
CD8+ T-CYT	Cytotoxic T Cell, CD8+	3.07	1.28E-09
aEC-GC	Glomerular Capillary Endothelial Cell	1.87	0.00000527

Supplemental Figure 12. Glomerular endothelial cells express *IL27RA* in human CKD kidneys. The Kidney Precision Medicine Project tissue atlas was interrogated for expression of IL27RA in CKD patient samples **(A)** UMAP plot showing cell populations with **(B)** expression of *IL27RA*. **(C)** Table showing cell clusters with significant upregulation and expression of *IL27RA* in human CKD kidneys (compared to other clusters, arrows point to glomerular capillary endothelial cells).

Supplemental Figure 13

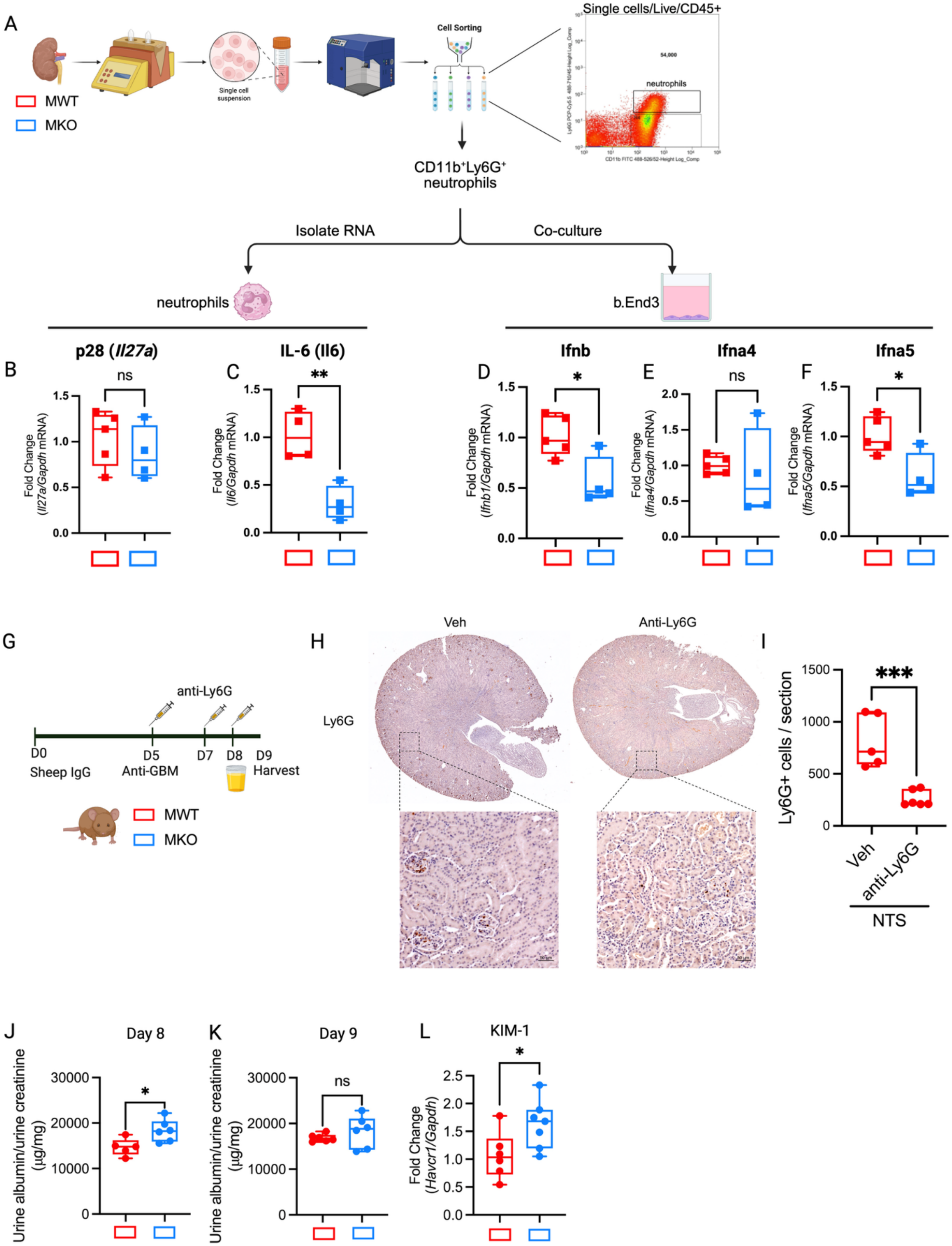

Supplemental Figure 13. Neutrophils are not the primary drivers of the enhanced glomerular and tubule injury phenotype observed in MKO mice during NTS nephritis. (A) Experimental workflow. Neutrophils ($CD11b^{+}Ly6G^{+}$) were isolated from the kidneys of MWT and MKO mice following NTS nephritis and analyzed directly or co-cultured with mouse endothelial cells (b.End3). Gene expression was subsequently assessed by quantitative PCR. **(B-C)** Relative mRNA expression of p28 (*Il27a*) and *Il6* in sorted neutrophils from MWT and MKO mice shown in boxplots (n = 4-5; *p< 0.05, **p< 0.01 by unpaired T test). **(D-F)** Boxplots show expression of type I IFN genes (*Ifnb, Ifna4, Ifna5*) in bEnd.3 cells following co-culture with neutrophils isolated from MWT or MKO mice (n = 4-5; *p< 0.05 by unpaired T test). **(G)** MWT and MKO mice were subjected to NTS induction and randomly assigned to receive vehicle or anti-Ly6G antibody treatment. Neutrophils were depleted by anti-Ly6G injections (200µg/injection) on day 5, day 7 and day 8. Urine was collected on Day 8, and mice were harvested on day 9. **(H)** Representative Ly6G immunohistochemistry (IHC) staining of kidney sections from NTS mice treated with vehicle (Veh) or anti-Ly6G antibody. Enlarged images of the boxed regions are shown below. **(I)** Boxplot shows quantification of Ly6G+ cells per kidney section. Each dot represents one mouse (n= 5-6/group, ***p<0.001 by unpaired T test). **(J-K)** Urinary albumin excretion was measured and normalized to urinary creatinine on **(J)** day 8 and **(K)** day 9. Each dot in boxplot represents one mouse (n=6-7/group; *p<0.05 by determined by unpaired T test). **(L)** Kidney mRNA levels of kidney injury marker KIM-1/*Havcr1* was measured. Graphs display boxplot with dots indicating individual samples (n=6-7/groups; *p<0.05 by unpaired T test). Circles represent female animals and squares represent male animals. Created with Biorender.com.

Supplemental Figure 14

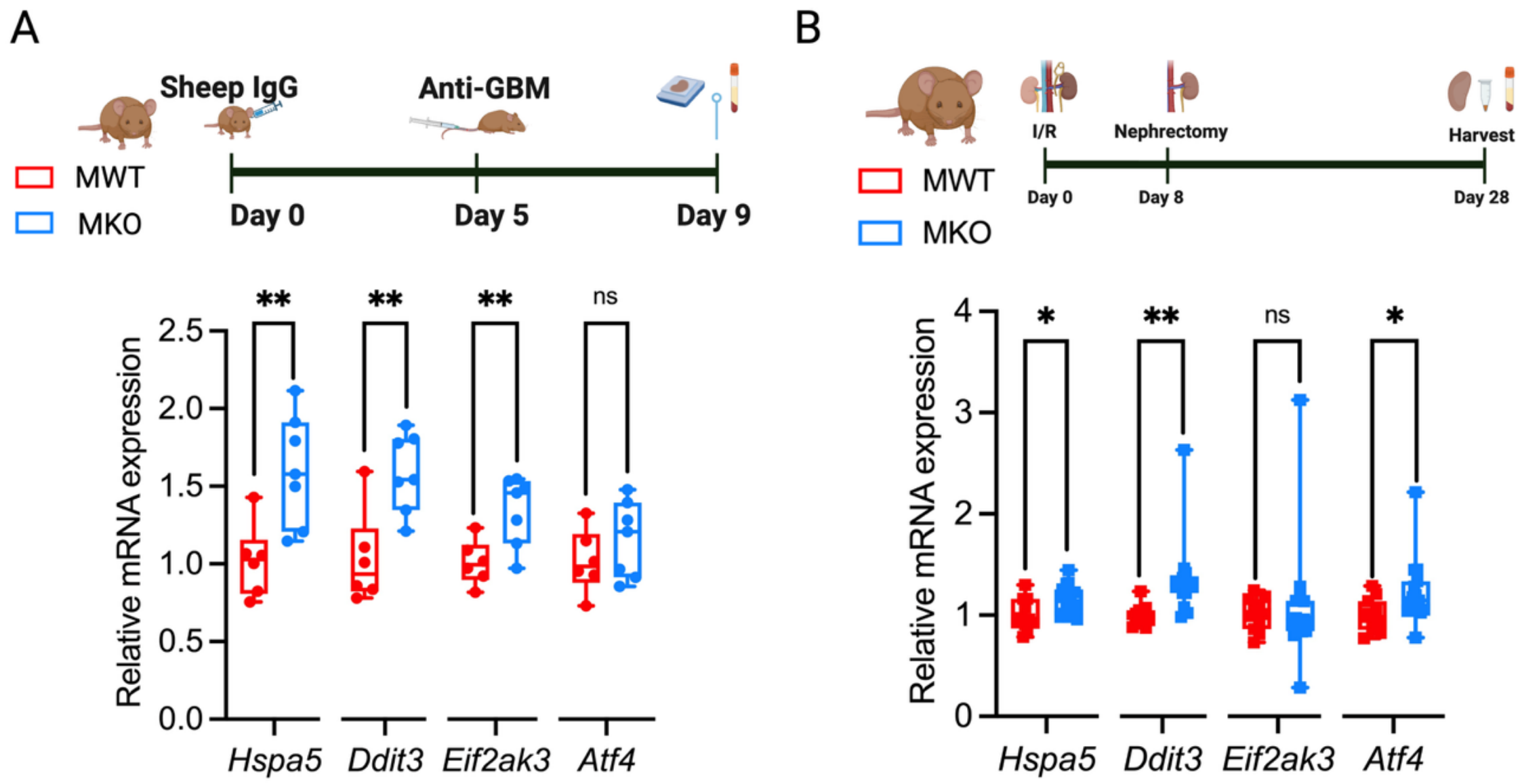

Supplemental Figure 14. ER stress marker expression in whole kidneys following NTS nephritis and chronic ischemia-reperfusion injury. (A) Experimental schematic of the nephrotoxic serum nephritis (NTS) model. IL1R MWT and MKO mice were subjected to NTS induction. Kidneys were harvested on day 9. RT-PCR was performed for mRNA levels of ER stress-related genes *Hspa5* (BiP), *Ddit3* (CHOP), *Eif2ak3* (PERK), and *Atf4* in whole kidney lysates (n = 6-7/group; **p < 0.01 by unpaired T tests). **(B)** Experimental schematic of the chronic ischemia-reperfusion injury (I/R) model. IL-1R1 MWT and MKO mice underwent unilateral renal ischemia-reperfusion injury on day 0 followed by contralateral nephrectomy on day 8. Kidneys were harvested on day 28. RT-PCR was performed for mRNA levels of *Hspa5* (BiP), *Ddit3* (CHOP), *Eif2ak3* (PERK), and *Atf4* in whole kidney lysates (n = 11-15/group; *p < 0.05, **p < 0.01 by unpaired T tests). Graphs display boxplots with dots indicating individual mice. Schematics created with Biorender.com. Circles represent female animals and squares represent male animals.

Supplemental Table 1.

**Supplemental Table 1: Primers used for Taqman RT-PCR analysis**	
**Gene**	**Accession number/catalog number**
Gapdh	Mm99999915_g1
Havcr1	Mm00506686_m1
Lcn2	Mm01324470_m1
Ifnb1	Mm00439552_s1
Ifna4	Mm00833969_s1
Ifna5	Mm00833976_s1
Hspa5	Mm00517691_m1
Ddit3	Mm01135937_g1
Eif2ak3	Mm00438700_m1
Atf4	Mm00515325_g1
Il1r1	Mm00434235_m1
Il6	Mm01210732_g1
Tgfb	Mm01178820_m1
Fn	Mm01256744_m1
Il1rn	Mm00446186-m1
Ebi3	Mm00469294_m1
p28	Mm00461162_m1
p35	Mm00434169_m1

Supplemental Table 2.

**Supplemental Table 2: Fluorescent cell sorting antibodies**			
**Antibody**	**Company**	**Catalog number, RRID**	**μl/test**
Anti-CD16/CD32	BioLegend	101320, RRID:AB_1574973	1.5
FITC anti-CD11b	BioLegend	101206, RRID:AB_312789	0.25
PerCP/Cy5.5 anti-Ly6G	BioLegend	127616, RRID:AB_1877272	0.25
PE anti-CD11c	BioLegend	117308, RRID:AB_313777	0.25
PE-Cy7 anti-F480	BioLegend	123114, RRID:AB_893490	1
APC anti-CD64	BioLegend	139306, RRID:AB_11219391	1
BV421 anti-CD103	BioLegend	121422, RRID:AB_2562901	1
BV510 anti-Ly6C	BioLegend	128033, RRID:AB_2562351	0.5
Super Bright 645 anti-MHCII	ThermoFisher	64-5321-82, RRID: AB_2662402	0.25
BV785 anti-CD45	BioLegend	103149, RRID:AB_2564590	0.1
PerCP/Cy5.5 anti-CD31	Biolegend	102522, RRID:AB_2566761	0.25
PE anti-CD140b	Biolegend	136005, RRID:AB_1953270	0.5
APC anti-CD90.2	Biolegend	105311, RRID:AB_313182	0.25
BV711 anti-CD4	Biolegend	100550, RRID:AB_2562099	0.125
Percp/Cy5.5 anti-CD8	Biolegend	100734, RRID:AB_2075238	0.25
BV785 anti-IFNγ	Biolegend	505838, RRID:AB_2629667	1
PE anti-Foxp3	eBioscience	12-5773-80, RRID:AB_465935	1
APC anti-IL17A	Invitrogen	17-7177-81, RRID:AB_763580	1
PE-Cy7 anti-IL4	Biolegend	504117, RRID:AB_10895747	1
