# Supplementary material for: The myeloid IL-1 receptor limits IL-27–mediated endothelial type I IFN during nephrotoxic serum nephritis
Source: JCI Insight. 2026 Sep 1;11(19):e200728. doi: 10.1172/jci.insight.200728 (PMC13726424; doi:10.1172/jci.insight.200728)
Supplement: Supplementary file 2 [file jciinsight-11-200728-s183.pdf]

Full unedited blot for figure 2C_IRF7

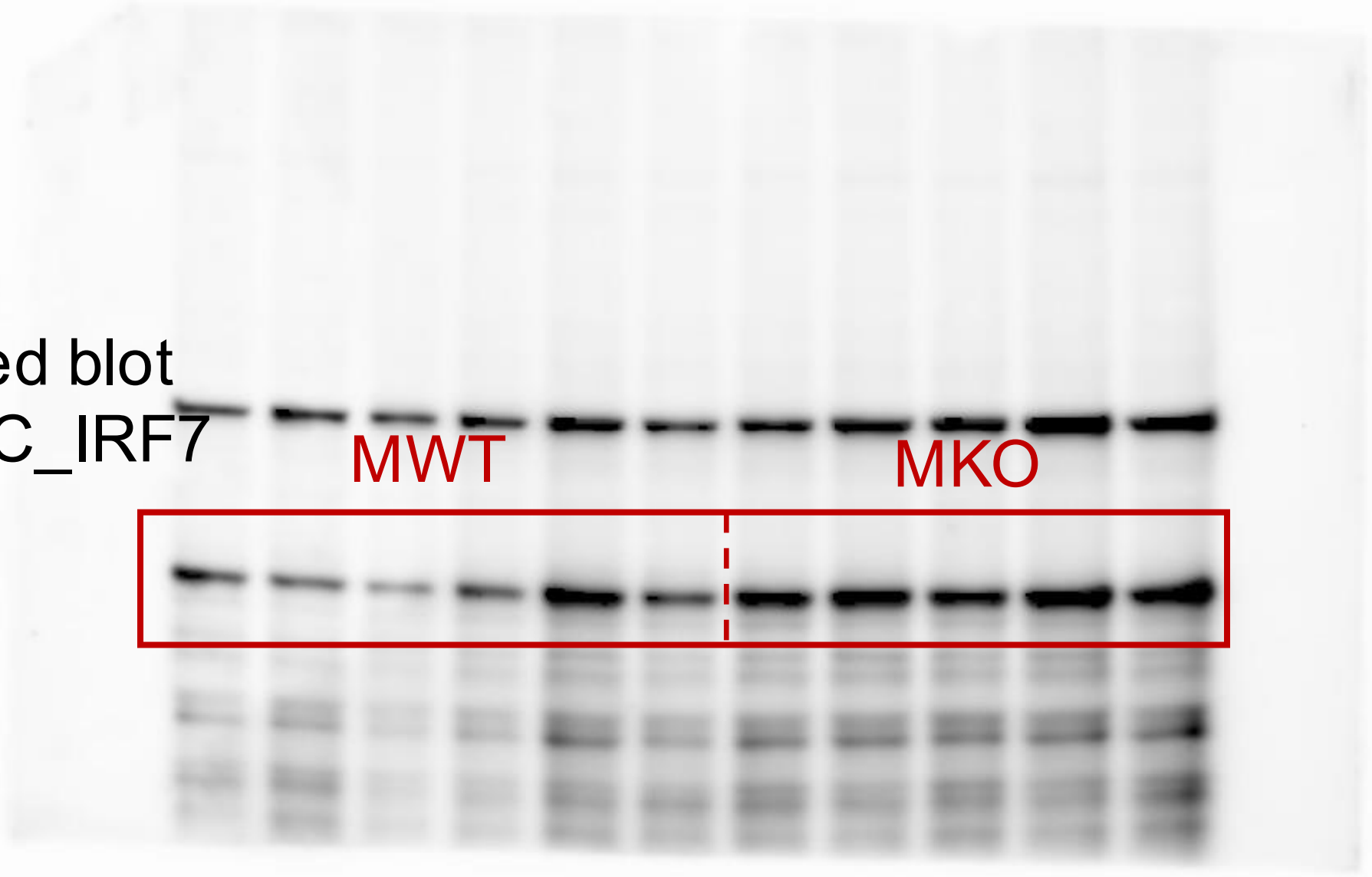

Full unedited blot for figure 2C_Histone3

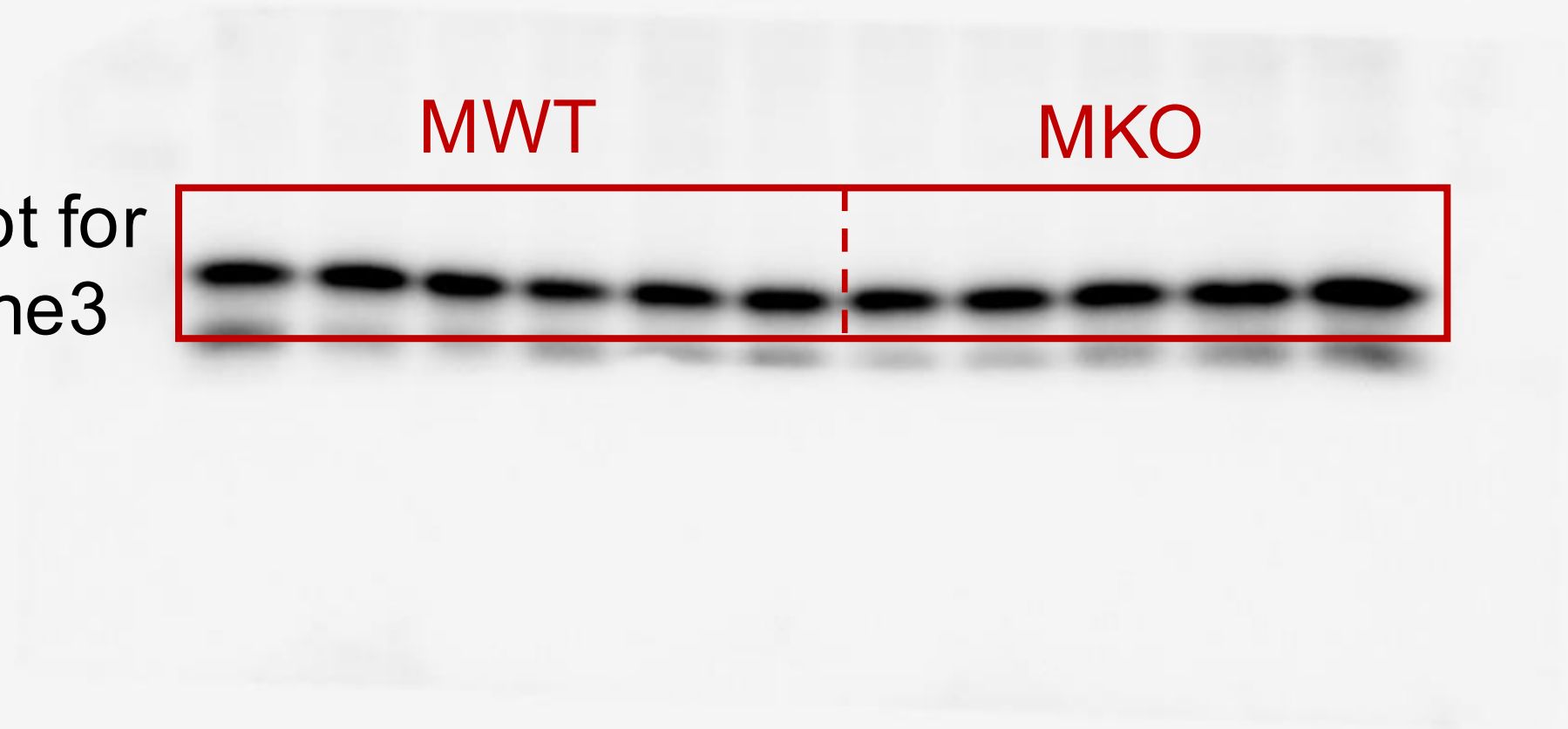

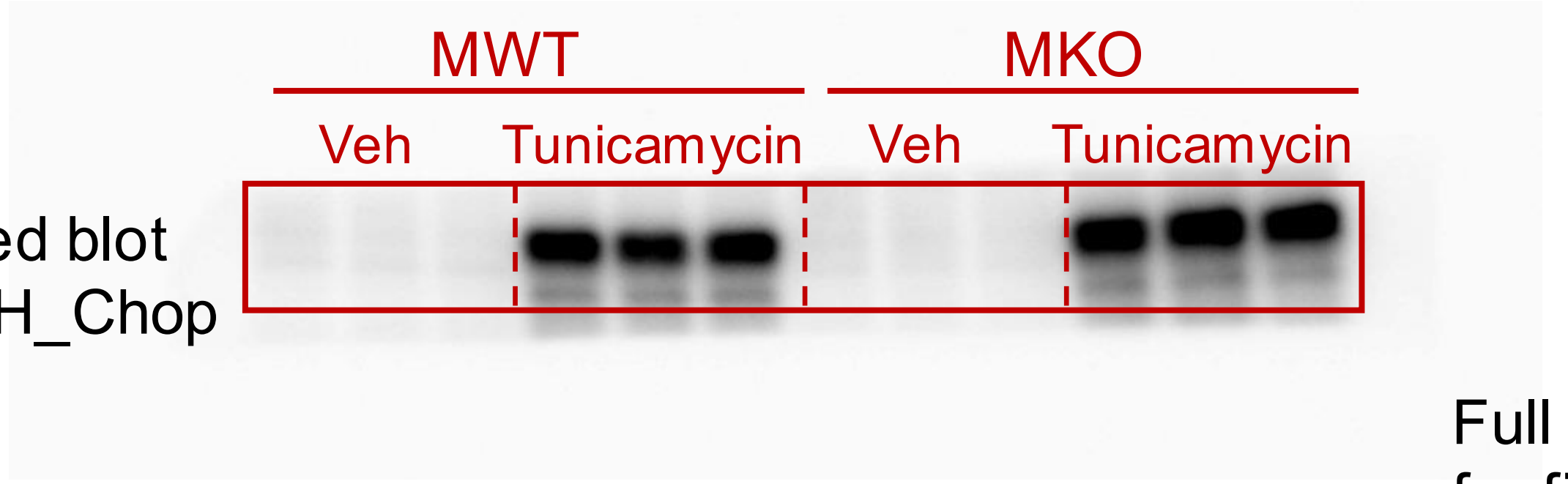

Full unedited blot for figure 5H_Chop

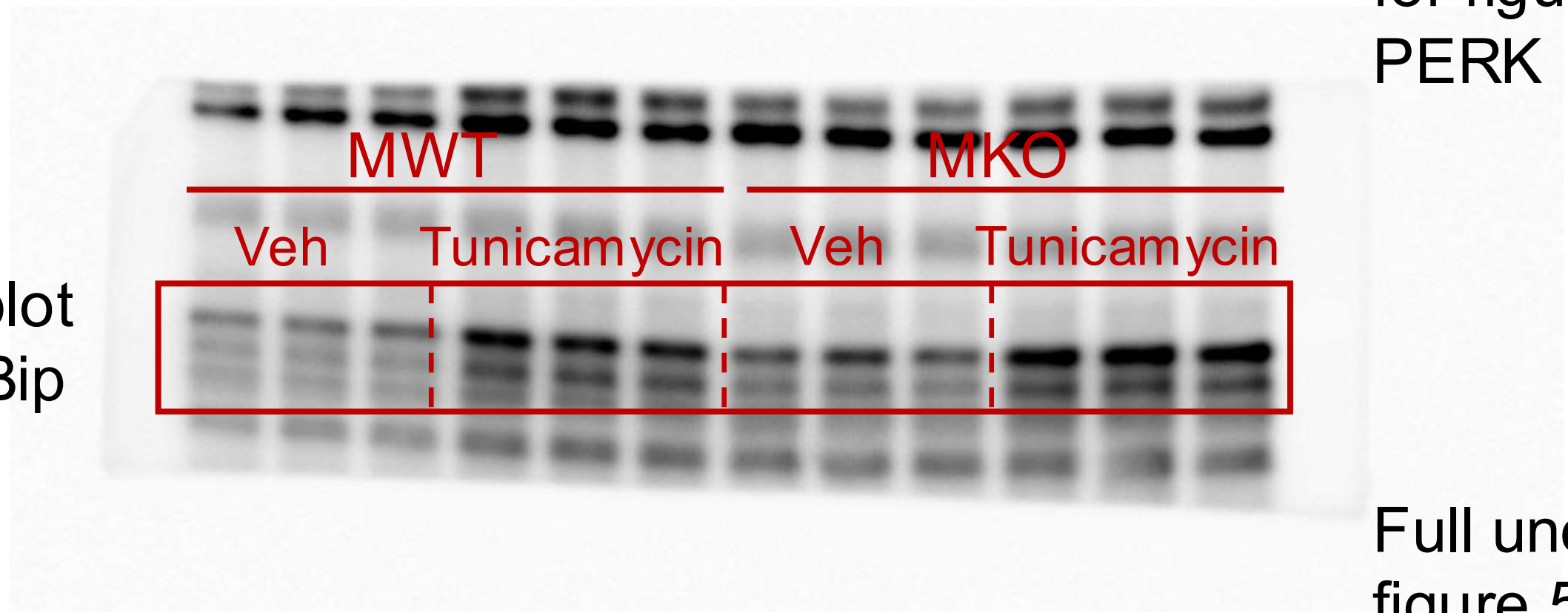

Full unedited blot for figure 5H_Bip

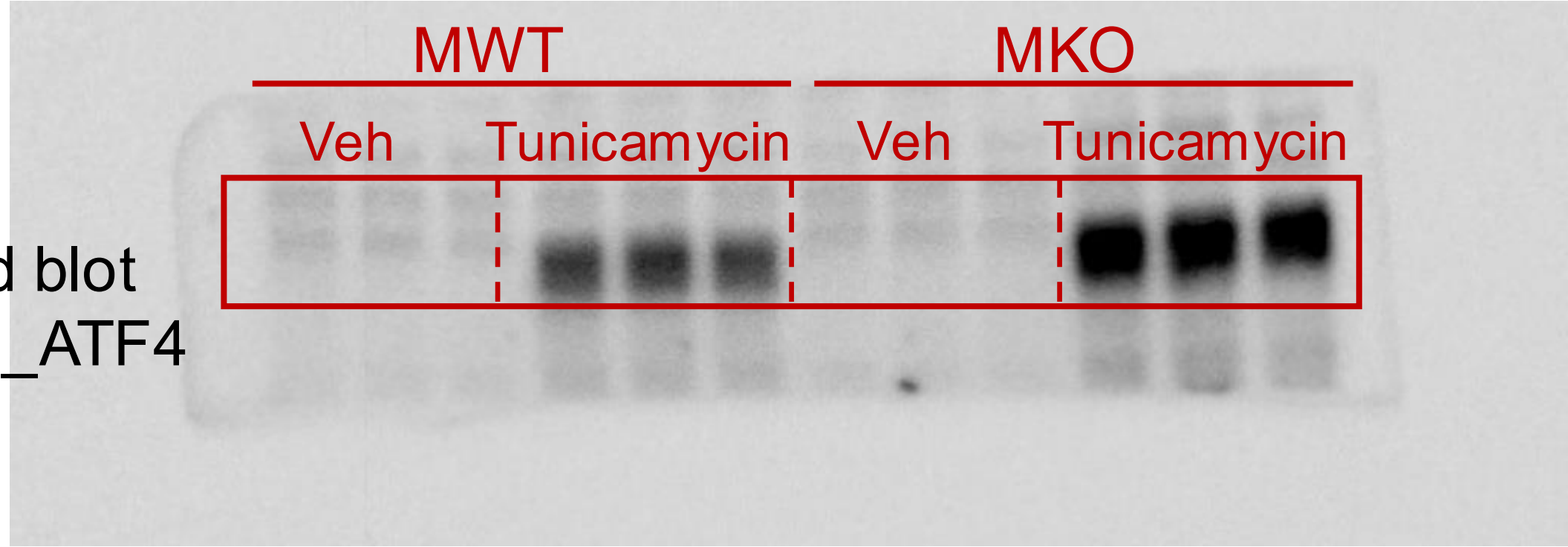

Full unedited blot for figure 5H_ATF4

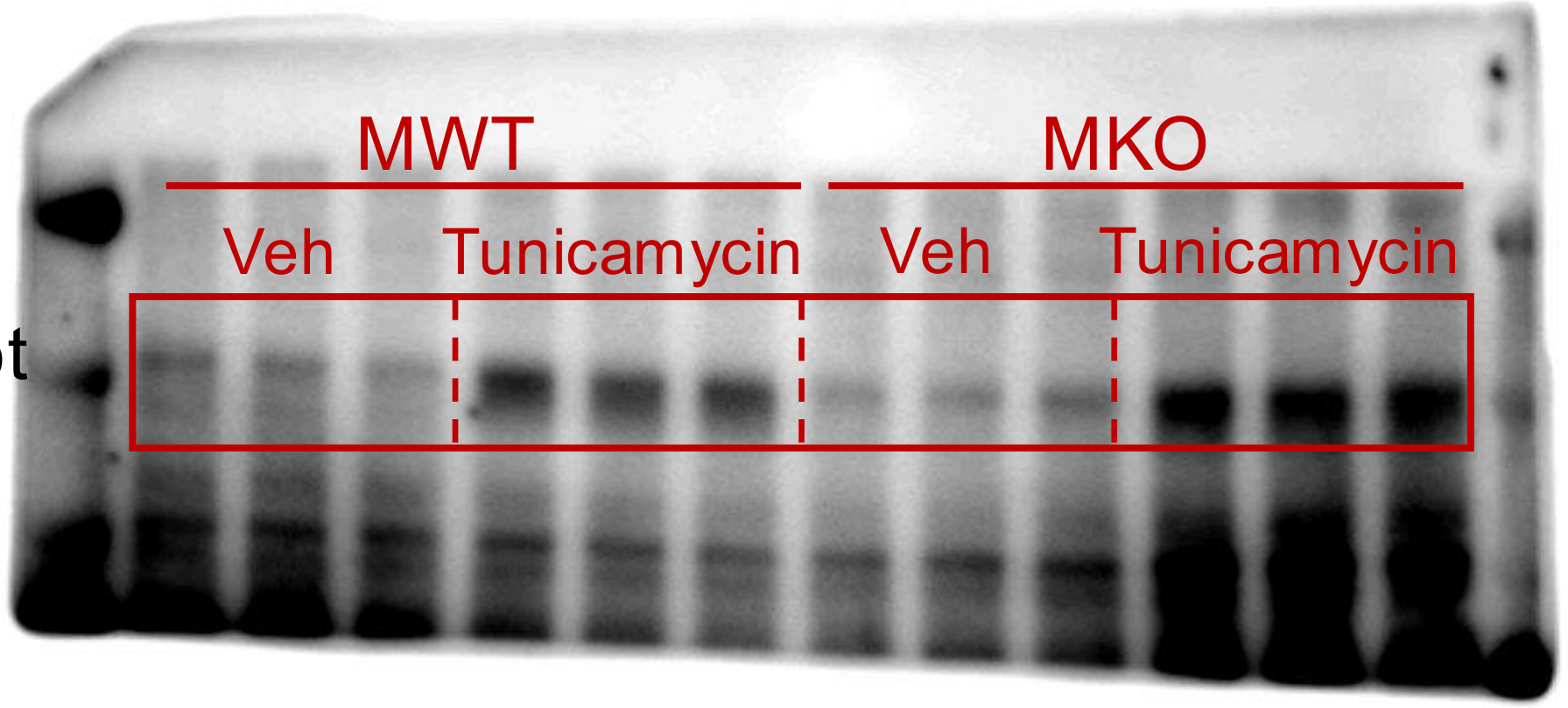

Full unedited blot for figure 5H_p-PERK

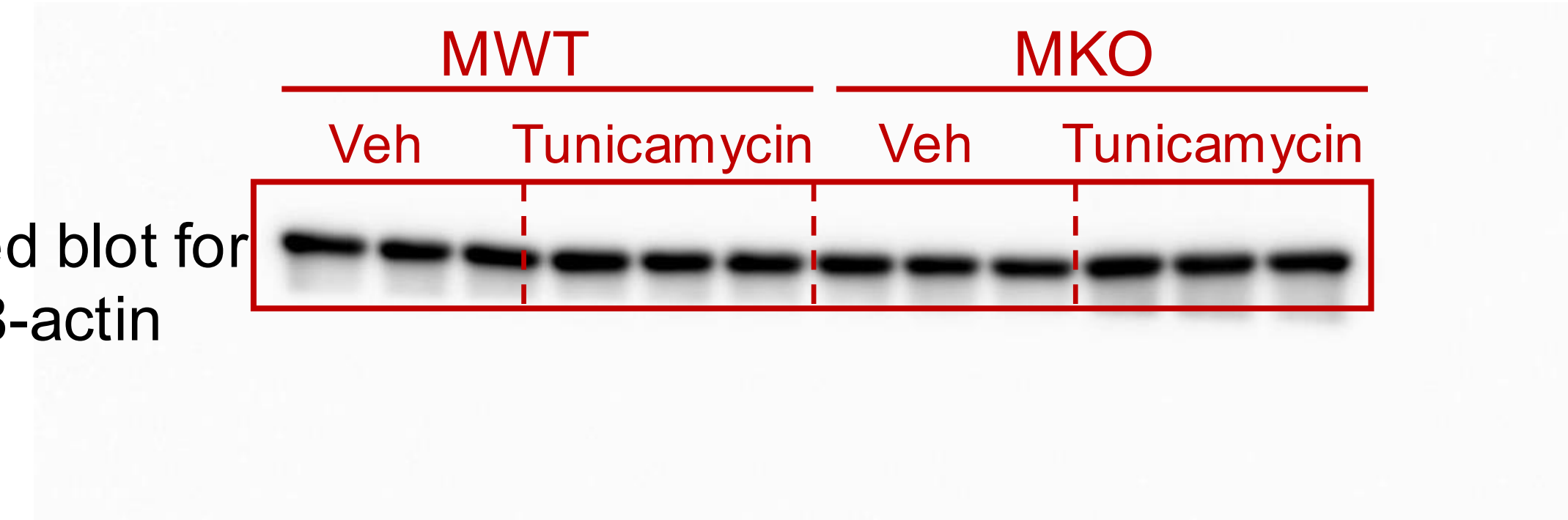

Full unedited blot for figure 5H_β-actin
